# 20-hydroxyecdysone promotes brain development via upregulating MMP2 expression during metamorphosis in *Helicoverpa armigera*

**DOI:** 10.1371/journal.pgen.1012032

**Published:** 2026-01-22

**Authors:** Can Tian, Pei-Yao Feng, Lin Wang, Tian-Wen Liu, Yan-Xue Li, Xiao-Fan Zhao

**Affiliations:** Shandong Provincial Key Laboratory of Development and Regeneration, School of Life Sciences, Shandong University, Qingdao, China; Indian Institute of Science Education and Research Mohali, INDIA

## Abstract

Matrix metalloproteinases (MMPs) play crucial roles in both physiological and pathological conditions by degrading the extracellular matrix; however, the roles and regulatory mechanisms of MMPs in brain development remain insufficiently understood. In this study, using the lepidopteran insect *Helicoverpa armigera*, the cotton bollworm, a serious agricultural pest, as an experimental model, we revealed that MMP2 is an important factor in insect brain development during metamorphosis under steroid hormone 20-hydroxyecdysone (20E) regulation. MMP2 is highly expressed in the brain during metamorphosis. MMP2 is localized in some surface and internal cells in the brain during metamorphosis. The knockdown of *Mmp2* by RNA interference in larvae repressed brain development, accompanied by an increase in autophagy and a decrease in cell proliferation. In addition, the nutrient levels of glucose and glutamate decreased in the brain, and the expression of glucose transporters and glutamate transporters decreased after *Mmp2* was knocked down. The transcription of *Mmp2* was upregulated by 20E via the transcription factor forkhead box O (FOXO) in a time- and concentration-dependent manner. These data suggest that MMP2 facilitates neural cell proliferation and nutrient supply, and ultimately regulates brain development during insect metamorphosis.

## Author summary

This study explores the role of matrix metalloproteinase 2 (MMP2) in brain postembryonic development during metamorphosis in the cotton bollworm, *Helicoverpa armigera*. Researchers found that MMP2, highly expressed in the brain during metamorphosis under the regulation of the steroid hormone 20-hydroxyecdysone (20E), is crucial for brain development. MMP2 facilitates neural cell proliferation and the accompanying expression of nutrient transporters, thereby ensuring a stable nutrient supply. Knockdown of *Mmp2* repressed brain development, reduced nutrient levels (glucose and glutamate), and increased neural cell autophagy, highlighting its roles in regulating neural cell proliferation and maintaining nutrient supply. The study also revealed that 20E upregulates MMP2 expression through the transcription factor FOXO, linking steroid hormonal signaling to brain development. These findings provide insights into postembryonic brain development and may offer new targets for pest control by disrupting metamorphosis. The research underscores the importance of MMP2 in coordinating brain postembryonic development and nutrient dynamics during insect metamorphosis under 20E regulation.

## Introduction

Matrix metalloproteinases (MMPs) are protease hydrolases capable of degrading almost all extracellular matrix (ECM) components [1]. MMPs are widely distributed and play roles in many biological processes, such as tissue remodeling and growth, trauma repair, and tumor transformation [2]. In *Drosophila melanogaster*, MMPs have been shown as key extracellular proteases involved in tissue remodeling during metamorphosis to digest type IV collagen, fibronectin, laminin, and cadherin [3]. *Drosophila* MMP2 regulates ovulation, wound healing, tissue remodeling, and follicle stem cell proliferation [4]. *Drosophila* MMP1 mutants cause defects in pupal head eversion and larval tissue histolysis during metamorphosis [5]. MMP in the lepidopteran *Galleria mellonella* during metamorphosis causes degradation of collagen-IV [6].

MMPs also play important roles in the mammalian nervous system and participate in generating various neurological diseases through their ECM degradation characteristics [7]. MMPs are associated with blood-brain barrier (BBB) breakdown and basal lamina type IV collagen degradation in human ischemic stroke [8]. During a stroke, MMPs are secreted by endothelial cells of the BBB to degrade the basement membrane of the BBB and neurovascular tissues to increase permeability and lead to neuronal damage and neuroinflammation [9]. MMPs are also secreted by astrocytes and neurons to induce BBB destruction [10]. MMPs allow adult visual cortex plasticity in mice by degrading the ECM [11]. These studies suggest MMPs play roles in the brain by their ECM degradation characteristics to increase the permeability and plasticity of the brain.

MMPs are regulated at several levels, including the activation of zymogens, the inhibitory effect of endogenous tissue inhibitors of matrix metalloproteinases, cellular localization, and gene expression [2]. Transforming growth factor beta (TGF-β) and alpha (TNF-α) promote MMP-9 expression in breast cancer metastasis [12]. The transcription factor activator protein-1 (AP-1) and constitutive transcription factor (SP1) can bind to the promoter regions of MMP genes to promote their transcription [13]. Although many studies have focused on MMPs, other biological aspects of the roles of MMPs in brain postembryonic development, especially the regulatory mechanism of MMP expression in the brain by a steroid hormone, are still unclear.

Brain growth requires a high nutrient supply, and most nutrients need transporters to be sent into the brain from the circulatory system, including blood in mammals and hemolymph in insects, because neural cells are highly differentiated cell types and lose the ability to synthesize some kinds of nutrients. The large molecules cannot cross the BBB and need transporters to enter the brain. For example, glucose needs to be transported into the brain by sodium-independent glucose transporters (GLUTs), encoded by the solute carrier family 2 (SLC2) genes [14,15]. However, glutamate is considered to be produced in neural cells and not to be transported into the brain from the circulatory system in mammals [16]. The regulation of the nutrient dynamics in the brain is not well understood.

The insect brain undergoes remodeling during metamorphosis [17]. The insect brain not only substantially changes in morphology and size but also shows neural cell death and proliferation during metamorphic development [18]. The adult nervous system is made up of remodeled larval neurons and neurons that are produced during metamorphosis by neuroblasts that persist from the embryonic stage [19,20]. The energy supply in the brain after the feeding stop during metamorphosis is an intriguing question. 20-hydroxyecdysone (20E) is a steroid hormone that is the major hormone to promote tissue remodeling during insect metamorphosis [21]. 20E also participates in many neural events, such as neuronal remodeling, programmed cell death, adult neuronal growth, and differentiation [18]. In addition, a previous study reported that hemolymph glucose levels are increased [22] via gluconeogenesis using the substrates produced by fat body autophagy and apoptosis under 20E regulation [23]. The glutamate levels are also increased in the insect hemolymph during metamorphosis [24]; however, the importance and transport mechanism of the increased hemolymph glucose and glutamate levels for the development of the brain during metamorphosis after the end of feeding are unclear. The insect brain is a good model for studying these questions in postembryonic brain development.

In this study, we used the cotton bollworm, a lepidopteran agricultural pest, *Helicoverpa armigera*, as a model to study the regulatory effect of the steroid hormone 20E on the transcription of MMP2 and the functional mechanism of MMP2 in brain postembryonic development during metamorphosis. The studies include screening the highly expressed MMPs in the brain during metamorphosis, analyzing the expression profiles of MMP2, localizing MMP2 in the brain, and exploring the roles of MMP2 in brain development during metamorphosis. The transcriptional regulation of MMP2 under 20E regulation was also determined by a luciferase reporter system. Through these experiments, we showed that 20E promotes MMP2 expression, which facilitates neural cell proliferation and nutrient transporter expression to increase the nutrient supply, therefore supporting brain development during insect metamorphosis. These findings provide new insights into animal brain postembryonic development and nutrient supply.

## Results

### Screening of MMPs that are highly expressed in the brain during metamorphosis

The MMPs were identified from the genome of *H. armigera* in the National Center for Biotechnology Information (NCBI, https://blast.ncbi.nlm.nih.gov/Blast.cgi) using the MMPs of *Homo sapiens*, *Mus musculus*, *D. melanogaster*, *Bombyx mori,* and *Aedes aegypti* as landmarks. Three MMPs were found in the genome of *H. armigera:* matrix metalloproteinase 2 (MMP2), matrix metalloproteinase 25 (MMP25), and matrix metalloproteinase 14 (MMP14) (S1 Fig). MMP2, MMP14, and MMP25 have similar domains; however, MMP2 has 2 transmembrane domains (S2 Fig). The sequence of MMP2 has 17.8% identity with that of MMP25 and 23.59% identity with that of MMP14 (S3 Fig). *Mmp2* was highly expressed in the brain of the 6th instar–96 h larvae (6th–96 h), *Mmp14* was highly expressed in the midgut of the 6th instar–96 h larvae, and *Mmp25* was highly expressed in the fat body of the 6th instar–96 h larvae (wandering stage of metamorphosis) compared with the 6th instar–24 h feeding larvae according to the *H. armigera* transcriptomic data (S4 Fig). qRT‒PCR confirmed that *Mmp2* was highly expressed in the brains of the 6th instar–96 h larvae, as determined using the primers in S1 Table (S5 Fig). Therefore, we used *Mmp2* as a target for further study.

### MMP2 has the highest expression levels in the brain during metamorphosis

**Fig 1:**
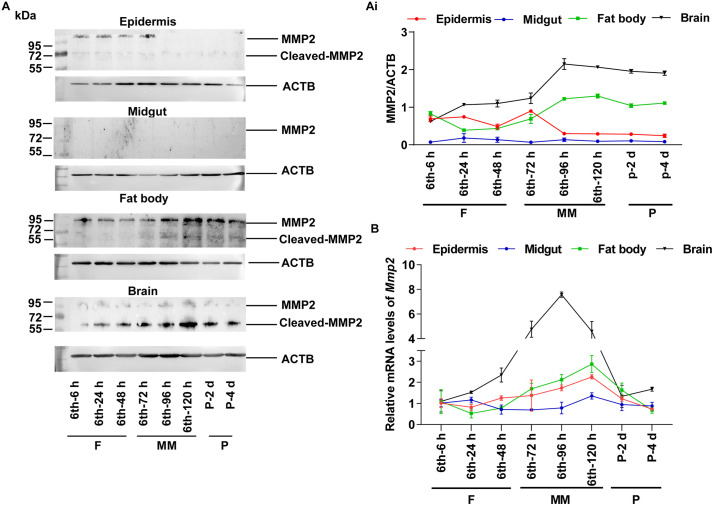
The expression profiles of MMP2 during development. **(A)** The protein levels of MMP2 in tissues at different developmental stages were measured via antibodies against MMP2. The secondary antibody used was horseradish peroxidase (HRP)-labeled sheep anti-rabbit IgG. ACTB was used as the internal reference protein. The concentration of the gel was 12.5%. (Ai) Quantification of the results of (A) by ImageJ. **(B)** qRT‒PCR was used to detect the mRNA levels of *Mmp2* in tissues. The sixth instar-6 h, sixth instar-24 h, sixth instar-48 h, sixth instar-72 h, sixth instar-96 h, and sixth instar-120 h correspond to different periods of the 6th instar, respectively. P-2 d and P-4 d: corresponding pupation days. F: feeding stage; MM: metamorphic molting stage; P: pupal stage. All the experiments were repeated three times. The bars indicate the means ± SD.

**Fig 2:**
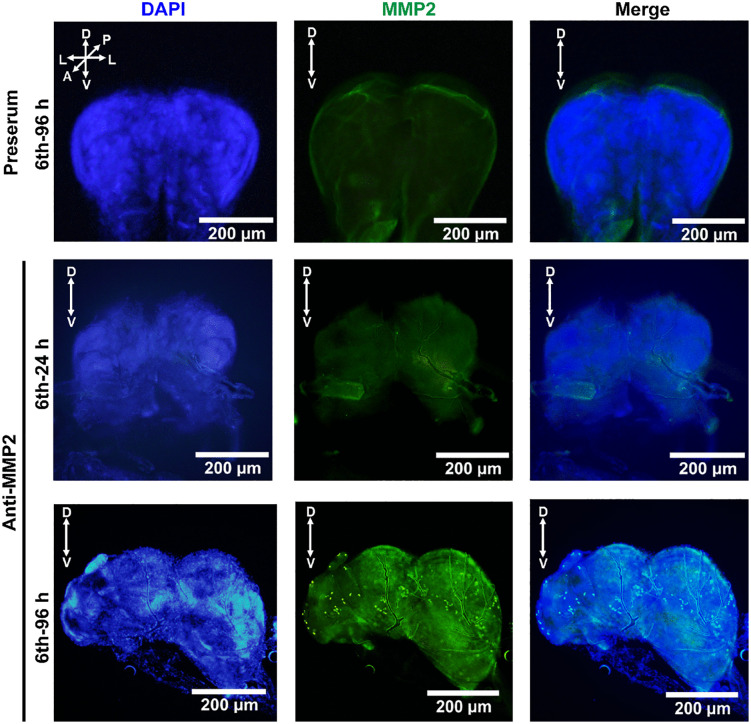
MMP2 is abundantly localized in the brain at 6th instar-96 h during metamorphosis, as shown by whole-brain immunohistochemistry. The preserum was used as a negative control. Green fluorescence: MMP2 was detected by rabbit polyclonal antibodies against *H. armigera* MMP2 and a secondary Alexa Fluor 488-conjugated sheep anti-rabbit IgG antibody. Blue fluorescence: DAPI. The picture was a whole-brain image. A, anterior; D, dorsal; L, lateral; P, posterior; V, ventral.

To verify the expression of MMP2 in the brain during metamorphosis, we determined the expression profiles of MMP2 in four tissues during development: the epidermis, midgut, fat body, and brain. Compared with those in other tissues, the expression levels of MMP2 in the brain were greater, and MMP2 was cleaved to its active form. MMP2 was also detected in the epidermis and fat body; however, MMP2 mainly appeared as a proenzyme. No MMP2 was detected in the midgut (Fig 1A and 1Ai) when rabbit polyclonal antibodies against *H. armigera* MMP2, which specifically recognize MMP2 (S6 Fig), were used. The qRT‒PCR results revealed similar expression profiles for *Mmp2* (Fig 1B). To determine the distribution of MMP2 in the brain, we detected MMP2 immunoreactivity in the brains of 6th instar-96 h larvae during metamorphosis. MMP2 was localized in some surface cells (putative BBB) and some inner cells of the brain by whole-mount immunohistochemistry (Fig 2) and single slide (S7 and S8 Figs), confirming the distribution of MMP2 in the brain during metamorphosis.

### Knockdown of *Mmp2* inhibited brain development

**Fig 3:**
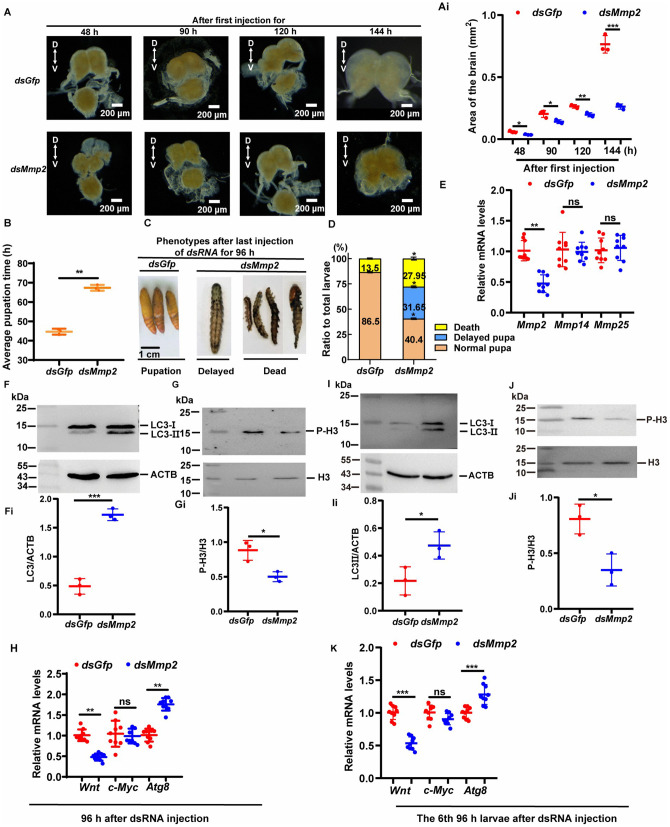
The knockdown of *Mmp2* repressed the development of the brain and pupation. **(A)** Morphology of the brain after injection of dsRNA for 48 h, 90 h, 120 h, or 144 **h.** D, dorsal; V, ventral. (Ai) Area of the brain after injection of dsRNA. n = 3. The bars indicate the means ± SD. Statistical analyses were conducted using Student′s *t* test (**, *p* < 0.01, ***, *p* < 0.001). **(B)** Average pupation time after injection of dsRNA. n = 30 × 3. The bars indicate the means ± SD. Statistical analyses were conducted using Student′s *t* tes*t* (**, *p* < 0.01). **(C)** Phenotypes after the injection of *dsGfp* or *dsMmp2*. **(D)** Statistical analysis of the phenotype after injection of *dsGfp* or *dsMmp2*. n = 30 × 3. The bars indicate the means ± SD. Statistical analyses were conducted using Student′s *t* test (*, *p* < 0.05). **(E)** qRT‒PCR was used *t*o detect the interference efficacy and off-target effects after the injection of *dsGfp* and *dsMmp2*. The bars indicate the means ± SD from three biological experiments and three technical repeats, which means the data were obtained from three preparations of cDNA, followed by three qRT-PCR repeats. **(F)** and **(G)** Knockdown of *Mmp2* caused autophagy and repressed the cell proliferation of the brain 96 h after dsRNA injection. LC3 and P-H3 were detected with rabbit polyclonal antibodies against LC3 and P-H3, respectively, and with secondary antibodies against horseradish peroxidase (HRP)-labeled sheep anti-rabbit IgG. (Fi) and (Gi) Quantitative statistics of the results of **(F)** and **(G)** graphs generated by ImageJ. Statistical analyses were conducted using Student′s *t* test (*, *p* < 0.05, ***, *p* < 0.001). The bars indicate the means ± SD from three biological experiments and three technical repeats. **(H)** Changes in the expression of *Wnt, c-Myc,* and *Atg8* (*Lc3*) of the brain 96 h after dsRNA injection were detected via qRT‒PCR. All the experiments were repeated three times. The bars indicate the means ± SD from three biological experiments and three technical repeats. Statistical analyses were conducted using Student′s *t* test (**, *p* < 0.01, ***, *p* < 0.001). **(I)** and **(J)** Knockdown of *Mmp2* caused autophagy and repressed the cell proliferation of the brain in the 6th-96 h larvae (developmental time was controlled by taking samples from the *Mmp2* knockdown larvae 20 h later than the control after dsRNA injection). LC3 and P-H3 were detected with rabbit polyclonal antibodies against LC3 and P-H3, respectively, and with secondary antibodies against horseradish peroxidase (HRP)-labeled sheep anti-rabbit IgG. (Ii) and (Ji) Statistics of the results of **(I)** and **(J)** graphs generated by ImageJ. Statistical analyses were conducted using Student′s *t* test (*, *p* < 0.05). **(K)** Changes in the expression of *Wnt, c-Myc,* and *Atg8* (*Lc3*) in the brain in the 6th-96 h larvae (developmental time was controlled by taking samples from the *Mmp2* knockdown larvae 20 h later than the control after dsRNA injection). The bars indicate the means ± SD from three biological experiments and three technical repeats. Statistical analyses were conducted using Student′s *t* test (***, *p* < 0.001).

To investigate the role of MMP2 in brain development, *Mmp2* was knocked down in 6th instar larvae by administration of *Mmp2* dsRNA into the hemolymph of 6th instar-6 h larvae. *Mmp2* knockdown repressed brain development, and the brain appeared smaller after *Mmp2* knockdown, compared with the control (Fig 3A and 3Ai). *Mmp2* knockdown resulted in delayed pupation for approximately 20 h (Fig 3B), 27.95% larval death, and a 31.65% delay in pupation for 20 h (Fig 3C and 3D). *Mmp2* was confirmed to be knocked down, and no reduction (off-target) was detected for the other two *Mmps* (Fig 3E), suggesting that MMP2 plays an important role in brain development and metamorphosis.

To investigate the molecular mechanism by which *Mmp2* knockdown slows brain development, we assessed autophagy and cell proliferation by examining autophagy-related protein 8 (ATG8/light chain 3, LC3) and phosphorylated histone H3 (P-H3), as these proteins are markers of autophagy and cell proliferation, respectively. The ATG8/LC3 protein is a widely used marker of autophagic vacuoles that is critical for autophagosome formation [25]. Phosphorylation at Ser10 (S10) of histone H3 is tightly correlated with chromosome condensation during both mitosis and meiosis [26]. The results revealed that after *Mmp2* was knocked down, there was an increase in the level of LC3-II (ATG8-II), suggesting that autophagy occurred in the brain (Fig 3F and 3Fi). In contrast, P-H3 was decreased by *Mmp2* knockdown (Fig 3G and 3Gi), suggesting that autophagy occurred and that cell proliferation was repressed by *Mmp2* knockdown.

To confirm this hypothesis, we examined the changes in the expression of *Wnt, c-Myc,* and *Atg8* because these genes are markers of cell proliferation and autophagy, respectively [25,27]. The results revealed that the expression of *Wnt* decreased, but the expression of *Atg8* increased (Fig 3H). These data indicate that *Mmp2* is necessary for brain development because it prevents autophagy and maintains cell proliferation in the brain. In these experiments, we detected the differences in genes and proteins in the larval brain 96 h after dsRNA injection without synchronizing the developmental time. The reason was that the difference in genes and proteins was produced once the *Mmp2* was knocked down, which caused the developmental delay.

To exclude the effect of delayed developmental time after *Mmp2* knockdown, we repeated the experiments by synchronizing the developmental time after *Mmp2* silencing by waiting for another 20 h to take samples from the *Mmp2* silencing group than the control group. Similar results were obtained, that LC3-II was increased, P-H3 was decreased, the expression of *Wnt* was decreased, but the expression of *Atg8* increased in the 6th-96 h larvae after *Mmp2* knockdown (Fig 3I-3K), which confirmed the above results.

### Knockdown of *Mmp2* resulted in a decrease in glucose and glutamate levels in the brain

**Fig 4:**
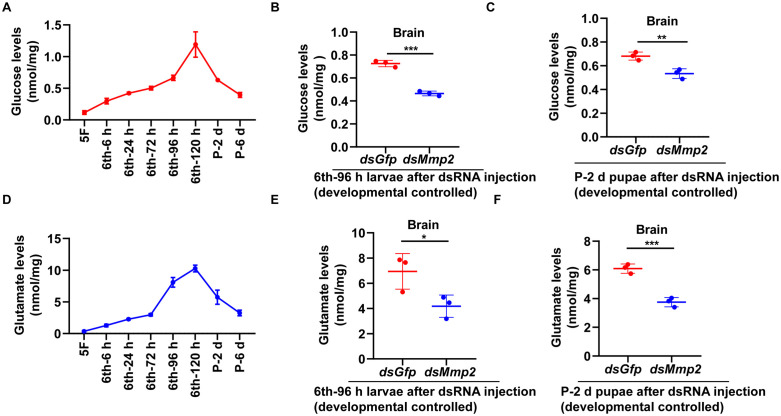
Glucose and glutamate levels in the brain. **(A)** Glucose levels in the brain at different developmental stages. **(B)** and **(C)** Glucose levels in the larval brain and pupal brain after the last injection of *dsMmp2* into the 6th-instar 6 h larval hemocoel. **(D)** Glutamate levels in the brain at different developmental stages. **(E)** and **(F)** Glutamate levels in the larval brain and pupal brain after the last injection of *dsMmp2* into the 6th-instar 6 h larval hemocoel. All the experiments were repeated three times. The bars indicate the means ± SD. Statistical analyses were conducted using Student′s *t* test (*, *p* < 0.05, **, *p* < 0.01, ***, *p* < 0.001).

To address the mechanism by which *Mmp2* knockdown induces a decrease in cell proliferation and an increase in autophagy in the brain, we examined the variation in the nutrient levels of glucose and glutamate in the brain. The results revealed that glucose levels increased in the brain during metamorphosis from 6th-72 h to P-6 d (Fig 4A). However, glucose levels decreased in the 6th-96 h larval brain and P-2 d pupal brain after *dsMmp2* injection (Fig 4B,4C). Similarly, glutamate levels increased in the brain during metamorphosis from 6th-72 h to P-6 d (Fig 4D). However, glutamate levels decreased in the 6th-96 h larval brain and P-2 d pupal brain after *dsMmp2* injection (Fig 4E and 4F). These data suggest that MMP2 is correlated with glucose and glutamate levels in the brain. In contrast, both glucose and glutamate levels increased in hemolymph after *Mmp2* was knocked down (S9 Fig). These results suggested that MMP2 regulated the levels of glucose and glutamate in the brain.

**Fig 5:**
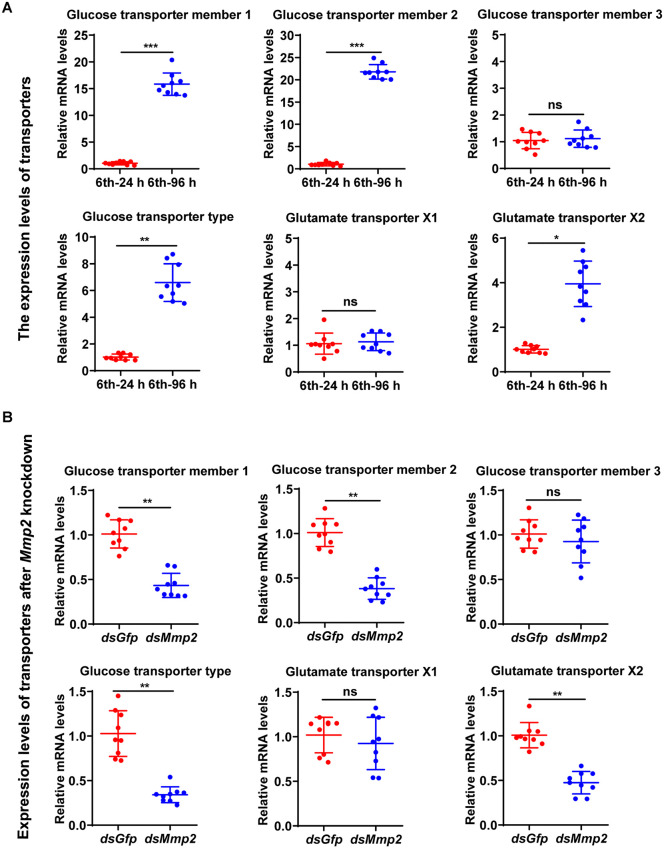
Relationship of the expression levels of transporters and *Mmp2* determined via qRT‒PCR. **(A)** The expression levels of glucose transporters and glutamate transporters in the brain during the feeding and metamorphic stages. The 6th instar-24 h was the feeding stage, and the 6th instar-96 h was the metamorphosis stage. **(B)** Expression levels of transporters in the brain after *Mmp2* knockdown. Transporter levels in the brain after the injection of *dsMmp2* into the 6th instar-6 h larval hemocoel for three days. All the experiments were repeated three times using three preparations of RNA and cDNA. The bars indicate the means ± SD. Statistical analyses were conducted using Student′s *t* test (*, *p* < 0.05, **, *p* < 0.01, ***, *p* < 0.001).

To explore the reason that glucose and glutamate levels decrease after *Mmp2* knockdown, the transporters of glucose (GLUT) and transporters of glutamate (GTs) were investigated. Four GLUTs, GLUT-1, GLUT-2, GLUT-3, and glucose transporter type (GLUT-t), and two GTs, GT-X1 and GT-X2, were found in the genome of cotton bollworms in the NCBI genome, and the relative expression levels in tissues were analyzed from the transcriptome database in the laboratory (S10 Fig). qRT‒PCR revealed that the expression of *Glut-1*, *Glut-2*, *Glut-t,* and *Gt-x2* increased during metamorphosis (Fig 5A), suggesting that these transporters are related to the decreases in glucose and glutamate levels caused by *Mmp2* knockdown.

To determine the relationship of the transporters with MMP2, the expression of the transporters was examined after *Mmp2* was knocked down by RNAi. The results revealed that the mRNA levels of *Glut-1*, *Glut-2*, *Glut-t,* and *Gt-x2* decreased after *Mmp2* knockdown (Fig 5B). To exclude the effect of delayed developmental time after *Mmp2* knockdown, we repeated the experiment by controlling the developmental time after dsRNA injection by waiting for 20 h to take samples from the *Mmp2* silencing group. Similar results were obtained; the mRNA levels of *Glut-1*, *Glut-2*, *Glut-t,* and *Gt-x2* decreased after *Mmp2* knockdown (S11 Fig). These data revealed a correlation between the nutrient transporters and MMP2.

**Fig 6:**
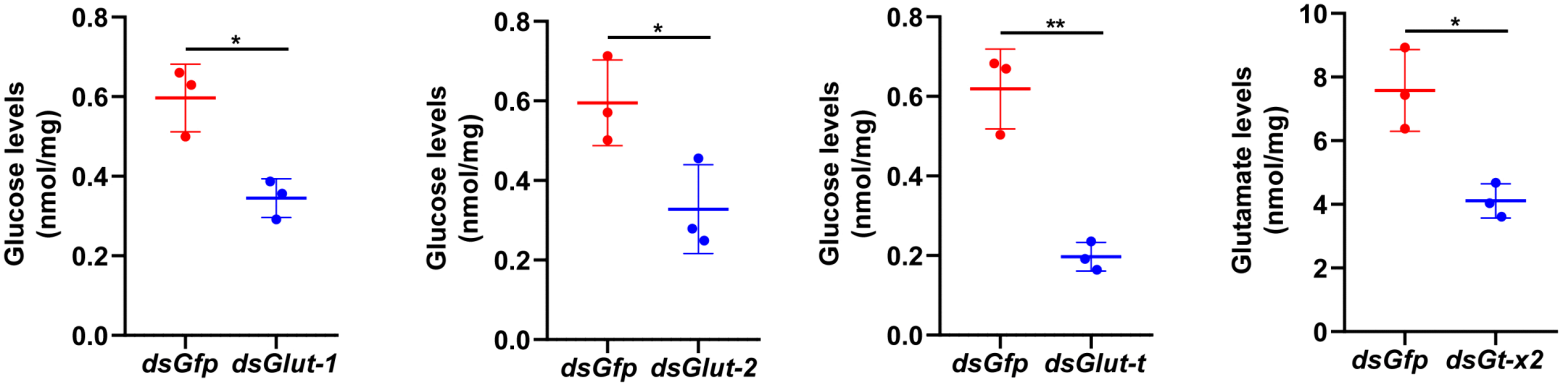
Nutrient levels in the brain after knockdown of their transporters in larvae. The nutrient levels were determined after last injection of dsRNA for three days. All experiments were repeated three times. The bars indicate the means ± SD. Statistical analyses were conducted using Student′s *t* test (*, *p* < 0.05, **, *p* < 0.01).

The transporters of glucose and glutamate were further knocked down to determine the relationship between nutrients and transporters in larvae. The results revealed that glucose was decreased in the brain after the knockdown of *Glut-1*, *Glut-2*, and *Glut-t*, and the glutamate levels decreased after the knockdown of *Gt-x2* (Fig 6)*.* These data confirmed the relationships between nutrient levels and transporters.

### *Mmp2* was upregulated by 20E

**Fig 7:**
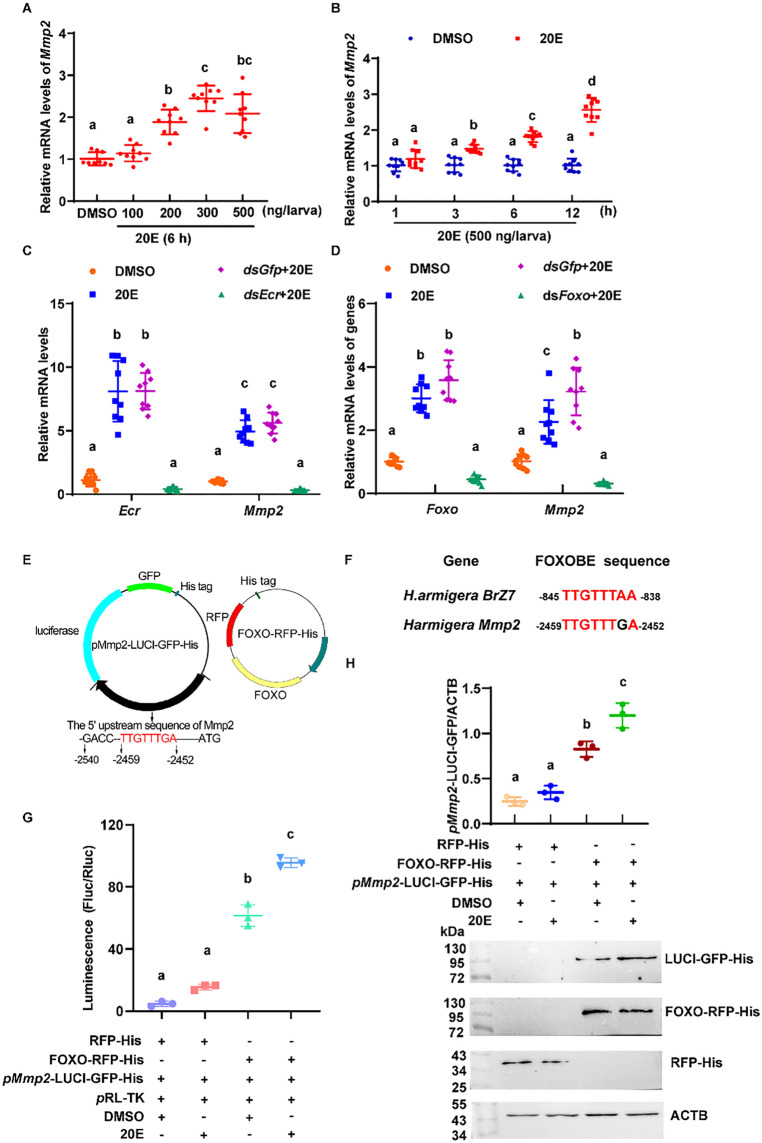
20E upregulates *Mmp2* expression. **(A)** qRT‒PCR was used to detect the expression level of *Mmp2* after injection of different concentrations of 20E for 6 h. The control group was injected with an equal amount of DMSO. **(B)** qRT‒PCR was used to detect the expression level of *Mmp2* at 1 h, 3 h, 6 h, and 12 h after the larvae were injected with 500 ng 20E/larva. The control group was injected with an equal amount of DMSO. The bars indicate the means ± SD. **(C)** qRT‒PCR was used to detect the expression level of *Mmp2* after *Ecr* was knocked down. **(D)** qRT‒PCR was used to detect the expression level of *Mmp2* after *Foxo* was knocked down. All the experiments were repeated three times using three preparations of RNA and cDNA. **(E)** Plasmid mapping of the *pMmp2-*LUCI-GFP-His and FOXO-RFP-His plasmids. **(F)** Sequence comparison of FOXOBE in the promoter regions of *H. armigera* Brz7 and *Mmp2*. **(G)** Dual-luciferase reporter assay to detect the transcriptional activity of the FOXOBE-containing sequence. **(H)** Western blot analysis of the expression of LUCI-GFP-His following FOXO overexpression under 20E regulation. The bands were statistically analyzed via ImageJ. All the experiments were repeated three times. The bars indicate the means ± SD. The data were analyzed by One-way ANOVA in A, and Two-way ANOVA in B to H, from three repeats for multiple comparisons with a Bonferroni correction. Different characters presented significant differences.

To demonstrate the regulatory mechanism of increased *Mmp2* expression during metamorphosis, 20E was injected into the hemolymph of 6th instar-6 h larvae to mimic high 20E levels during metamorphosis. The results revealed that the expression of *Mmp2* increased after treatment with 200–500 ng of 20E per larva for 6 h. Similarly, 20E significantly increased the expression of *Mmp2* after treatment with 500 ng of 20E for 3–12 h (Fig 7A, 7B), suggesting that 20E is a regulator of *Mmp2* expression.

To identify the transcription factor involved in 20E-mediated upregulation of *Mmp2* expression, the well-known transcription factors involved in 20E signaling, ecdysone receptor (*Ecr*) and forkhead box protein O (*Foxo*) [28], were knocked down in the HaEpi cell line. Knockdown of either *Ecr* or *Foxo* decreased 20E-induced *Mmp2* expression, suggesting that both EcR and FOXO participate in 20E-mediated upregulation of *Mmp2* expression (Fig 7C, 7D).

The 5′ upstream sequences of *Mmp2* were analyzed via the JASPAR website (https://jaspar.elixir.no/) to test this hypothesis. There is no EcR binding element predicted; however, a FOXO binding element (FOXOBE), TTGTTTGA, is conserved with FOXOBE in front of Br-Z7 [28]. Because *Foxo* is known to be regulated by EcR [28], therefore, FOXO regulating *Mmp2* expression was focused for further study*.* The *pMmp2*-LUCI-GFP-His reporter plasmid containing the FOXOBE and FOXO-RFP-His overexpression plasmid were constructed, respectively, and cotransfected into HaEpi cells (Fig 7E and 7F). A luciferase assay revealed that FOXO-RFP-His significantly increased the transcriptional activity of the *pMmp2*-LUCI-GFP-His reporter plasmid, and 20E enhanced the activity, compared with RFP-His control (Fig 7G), suggesting FOXO upregulated *Mmp2* transcription once it was overexpressed, because the overexpressed FOXO can automatically be localized in the nucleus [29]. However, the endogenous FOXO is localized both in the cytosol and nucleus, but enters the nucleus completely by 20E induction [28], which partially explains the increased transcriptional activity by 20E treatment. Immunoblotting experiments also revealed little expression of LUCI-GFP-His when the reporter plasmid was cotransfected with the RFP-His tag control; however, after the reporter plasmid was cotransfected with FOXO-RFP-His, the expression of LUCI-GFP-His was upregulated significantly in the DMSO control, and 20E increased the expression, compared with the RFP-His control (Fig 7H), confirming that FOXO upregulated *Mmp2* transcription. These data showed that 20E via FOXO upregulates *Mmp2* transcription.

## Discussion

The regulatory mechanism of insect brain development during metamorphosis remains incompletely understood. We revealed that the steroid hormone 20E can promote the expression of MMP2 through the transcription factor FOXO to facilitate neural cell proliferation and increase nutrient transporter expression to supply nutrients for brain development during insect metamorphosis.

### MMP2 facilitates neural cell proliferation

The size of the brain is increased during insect development. The number of neural cells substantially increases during insect metamorphic development from larval to adult stages [30]. Single-cell sequencing revealed that the cells of brain in first-instar larvae of *Drosophila* are clustered into five major cell populations, neural progenitors, differentiated neurons, glial cells, undifferentiated neurons, and non-neuronal cells [31], whereas the cells in adult *Drosophila* brain are clustered into 87 naïve cell groups, with approximately 231–1190 subcellular groups, including cholinergic neurons, glutamatergic neurons, GABAergic neurons, and glial cells that expressed also in larval brain [32]. There are approximately 100,000 neurons in the adult brain of *Drosophila*, which is two orders of magnitude greater than the number in the brain of *Drosophila* larvae [33]. The plasticity of the brain is essential for the major expansion of neural cell numbers and brain size. As a matrix metalloproteinase, MMP2 can degrade the ECM to increase the plasticity of the brain, thus guaranteeing cell proliferation. Part of the adult neural cells, the imaginal neural cells, are proliferated during metamorphosis from neuroblasts that persist from the embryonic stage. The neural cell proliferation is part of brain remodeling, which including PCD of larval neural cells and cell proliferation of adult neural cells, during metamorphosis. MMP2 facilitates neural cell proliferation by its proteolytic characteristics in brain remodeling during metamorphosis.

Evidence from mammalian systems indicates that MMPs can process signaling molecules (e.g., growth factors, cytokines) by liberating them from the ECM or cleaving cell surface precursors, thereby activating proliferative pathways [34,35]. In the context of insect brain development, we demonstrate that MMP2 is essential for neural cell proliferation. Knockdown of *Mmp2* significantly reduced phosphorylated histone H3 (P-H3) and *Wnt* expression (markers of proliferation), while increasing autophagy. These data establish a role for MMP2 in maintaining neural cell proliferation during postembryonic brain development. Further studies are warranted to determine whether MMP2 acts as an activator of the cell proliferation pathway in the insect brain.

### MMP2 is required for maintaining the nutrient supply

The brain is dependent on glucose as a fuel source [36]. Glucose is the primary energy source of the brain in mammals, which provides ATP for the brain by glycolysis and the TCA cycle (tricarboxylic acid cycle). Glucose must be transported into the brain from the blood by its transporter GLUT because the brain does not produce glucose as a final differentiated cell, and glucose cannot diffuse across cell membranes [37]. Endothelial cells of the BBB can express GLUT1 and GLUT3 in humans [14]. GLUT1 is also expressed in astrocytes to transport glucose into cells [38]. GLUT family members are facilitative transporters that transport glucose from high concentration to low concentration side without consuming energy [39]. Hemolymph glucose levels increase during metamorphosis via gluconeogenesis under 20E regulation in *H. armigera* [22]. The increased hemolymph glucose comes from the degradation of trehalose and glycogen, and gluconeogenesis by using the substrates of programmed cell death of the larval tissues after feeding stops during metamorphosis [23]. In this study, we revealed that glucose in the brain is increased during metamorphosis, too; however, the levels are lower than those in hemolymph. Knockdown of *Mmp2* decreased the glucose levels in the brain but increased the glucose levels in the hemolymph, suggesting the transportation of glucose into the brain from the hemolymph. Knockdown of *Mmp2* may repress cell proliferation and the expression of GLUTs, finally decreasing the transportation of glucose into the brain from the hemolymph. Furthermore, knockdown of *Gluts* decreased the glucose levels in the brain, suggesting that glucose is transported into the brain by GLUTs, which supply glucose to support brain development during metamorphosis.

Glutamate (Glu) is the main excitatory neurotransmitter in the central nervous system (CNS). Glu is synthesized de novo in astrocytes and neurons from glucose via α-ketoglutarate by the TCA cycle [40]. Glu can be converted from glutamine (Gln) by glutaminase in glutamatergic neurons, too. Glu in synaptic vesicles is released from neural cells as a neural transmitter and leads to a signal that plays roles in learning and memory [41]. Glutamate also promotes neural cell proliferation and migration by activating its receptors [41]. For example, glutamate released from glioblastoma cells promotes the migration of neighboring cells [42], and glutamate stimulates cell proliferation and increases the tumor mass [41]. Glutamate can also be used as a fuel to sustain neuronal activity [43]. Glutamate metabolism is shifted from glucose metabolism upon mitochondrial pyruvate carrier inhibition [44]. Glu and Gln can produce ATP by forming α-ketoglutarate and entering the TCA cycle [45]. However, excessive accumulation of glutamate in the extracellular fluid of the brain is toxic to neural cells because glutamate can induce calcium influx via the activation of glutamate receptors, which activate catabolic enzymes. Therefore, intracellular glutamate is well balanced by various glutamate transporters [41].

Multiple glutamate transporters have been identified, including the vesicular glutamate transporters (VGLUTs) and the sodium-dependent glutamate transporters within the solute carrier family 1 (SLC1), also known as excitatory amino acid transporters (EAATs) [46]. VGLUTs actively mediate the loading of glutamate into synaptic vesicles and facilitate its subsequent exocytotic release at the presynaptic terminal. EAATs actively transport glutamate from the synaptic cleft into glial cells to ensure its efficient clearance [47]. Glutamate is converted to glutamine in astrocytes by glutamine synthetase. Glutamine is exported via astrocytic sodium-coupled neutral amino acid transporters (SNATs) and imported into neurons to be reconverted to glutamate by glutaminase [45]. Part of the remaining glutamate will be converted to GABA, or α-ketoglutarate, by amino transferases in the cytoplasm or by glutamate dehydrogenase (GDH), an enzyme regulating the reversible reaction between α-ketoglutarate and glutamate, in the mitochondria to finally provide adenosine triphosphate (ATP) [47]. The transporter families are highly conserved between insects and mammals [48].

Glu cannot penetrate the BBB, as the studies [40]. The BBB is composed of cerebral endothelial cells in mammals and the perineurial and subperineurial glial cells in *D. melanogaster* [49]. Facilitative transport of glutamate, a type of energy-independent glutamate transport, is restricted to the luminal membranes of the endothelial cells of the BBB (blood lumen face). The active transporters, energy-dependent Na^+^-cotransporter EAATs (excitatory amino acid transporters) present only in the abluminal membrane of the BBB (brain side), can uptake glutamate from the extracellular fluids to prevent neuronal death induced by a calcium influx triggered by glutamate [50]. Therefore, glutamate may enter endothelial cells but cannot be transported into the brain from the endothelial cells [16], except in the circumventricular organs, there is no BBB [51]. Insects have an open circulatory system. Although they lack the vascular endothelium that forms the BBB in mammals, there are glial cells, including perineurial glial cells (PG) and subperineurial glia (SPG), between the insect brain and hemolymph that function as a BBB [52]. The PG forms the outer layer of the brain and is engaged in nutrient uptake. The SPG forms occluding septate junctions to prevent paracellular diffusion of macromolecules into the nervous system.

The concentration of Glu in the brain is much higher than in the blood in mammals [53]. A previous study reported that glutamate levels are increased in *H. armigera* hemolymph by larval tissue PCD during metamorphosis under 20E regulation to promote cell proliferation of the adult fat body [24]. This study further revealed an increase in glutamate in the brain during metamorphosis, and a parallel increase in the expression of glutamate transporter *Gt-x2*. As per the previous knowledge from mammals, Glu cannot be transported into the brain from blood; the increased Glu in the brain may come from the synthesis in neurons from glucose via the TCA cycle. Although we observed that knockdown of *Gt-x2* decreased glutamate levels in the brain, given that GT-X2 is a VGLUT, its reduction may disrupt synaptic glutamate packaging and recycling, indirectly perturbing overall glutamate homeostasis. Further studies are needed to validate whether this reflects impaired vesicular storage or altered synthesis or metabolism. VGLUTs in neurons or astrocytes not only uptake intracellular glutamate into vesicles, but also release the vesicles into the synaptic cleft via exocytosis [47]. Insect BBB in metabolic maintenance of the CNS requires further study [52]. In addition, we observed that knockdown of *Mmp2* decreased the glutamate levels in the brain but increased the glutamate levels in the hemolymph. The decrease of glutamate in the brain might be due to the repression of cell proliferation, which results in the decrease of glutamate synthesis in the brain. The mechanism of the increase in glutamate levels in the hemolymph after *Mmp2* knockdown requires further study. Our study agrees that the insect BBB (Blood-Brain Barrier) is a selective and flexible blood-brain interface in nutrient transport and signal communication [54].

The relationship between MMP2 and nutrient transporter expression remains to be elucidated. As a protease, MMP2 could not directly regulate transporter expression. While studies in mammalian systems indicate that MMPs can influence signaling pathways related to nutrient transport (e.g., MMP1 activates PI3K-AKT-mTOR signaling [55]), our data demonstrate only a correlative relationship: knockdown of *Mmp2* reduces glucose and glutamate levels and transporter expression in the insect brain. One possibility is that cell proliferation brings an increase in the expression levels of transporters. Another possibility is that 20E upregulates the transporter expression. In *Drosophila*, TGFβ ligand Gbb can induce the expression of the carbohydrate transporter Tret1–1 during metamorphosis [56]. The nutrient transporter expression under 20E regulation during metamorphosis needs future study.

In addition to the roles in transporting glucose and glutamate, via increasing cell proliferation, MMP2 may increase other transporters for other nutrients. MMP2 also plays roles in the integrity of structural components; all these effects account for brain development during metamorphosis. The effects of MMP2 on other transporters need further study. In addition, further study is needed to address the specific cell types of MMP2 and the transporters.

### 20E via FOXO upregulates MMP2 transcription

Although MMP2 is critical for tissue remodeling [57], most studies have focused on its activation. For example, MMP is cleaved by cathepsin L to cause fat body cell dissociation in *Helicoverpa armigera*, which activates lipid metabolism to result in energetic supplies and increase brain metabolic activity and thus promote pupal development. CRISPR-mediated gene knockout of *Mmp* delayed pupal development and increased the incidence of pupal diapause [58]. There are few studies on the transcriptional regulation of MMPs, especially by steroid hormones. Our study revealed that the steroid hormone 20E can promote the expression of *Mmp2* at the transcriptional level through the transcription factor FOXO, which provides a reference for the study of the regulation of MMPs in other organisms.

FOXO has been shown to play important roles in insect metamorphosis via 20E signaling. FOXO can be upregulated by 20E via the ecdysone nuclear receptor EcR; in turn, FOXO upregulates the expression of other genes in the 20E signaling pathway, including the Broad-complex isoform, which upregulates carboxypeptidase A expression for proteolysis during apolysis of insect molting [28]. FOXO upregulates the expression of autophagy-related genes for autophagy [59], and the expression of lipases for lipid degradation [60] during metamorphosis. FOXO is known as a negative regulator of cell proliferation; however, it plays roles in various cellular processes, including apoptosis and cell proliferation, by different mechanisms [61]. In humans, FOXO1 supports cancer cell proliferation [62]. This study presents evidence that FOXO upregulates MMP2 expression at the transcriptional level, and MMP2 is necessary for neural cell proliferation as an ECM-degrading enzyme; however, this does not mean that FOXO promotes cell proliferation directly.

## Conclusion

**Fig 8:**
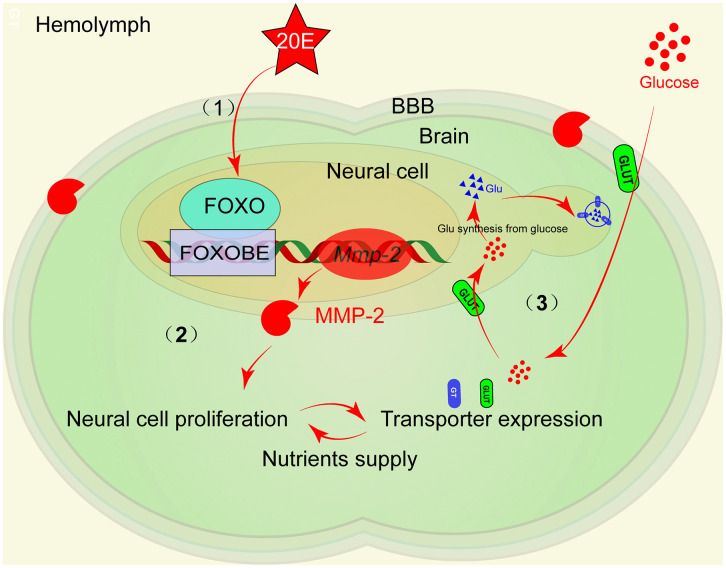
The mechanism of MMP2 expression and function in the brain. (1) 20E via FOXO promotes MMP2 expression. (2) MMP2 is required for neural cell proliferation. The expression of nutrient transporters is increased along with cell proliferation. (3) Nutrient transporters supply nutrients for brain development.

MMP2 exhibits high expression levels in the brain and is specifically localized to certain superficial and inner neural cells in the brain during insect metamorphosis. MMP2 facilitates imaginal neural cell proliferation during brain development from the larval brain to the adult brain by its proteolytic characteristics, which present plasticity of the brain. Cell proliferation brings an increase in the expression of nutrient transporters to ensure adequate nutrient supply for brain development during metamorphosis. Sufficient nutrient supply prevents neural cells from undergoing autophagy. The steroid hormone 20E upregulates MMP2 expression via the transcription factor FOXO. Together, 20E coordinately upregulates MMP2 expression, neural cell proliferation, and nutrient dynamics (Fig 8).

### Experimental animals and methods

#### Ethics statement.

The antibody preparation for rabbits was performed according to protocols approved by the Animal Care & Welfare Committee, Shandong University School of Life Sciences (SYDWLL-2021–59).

#### Experimental animals.

*H. armigera* were cultured in our laboratory at 27 ± 1°C under a photoperiod of 14 h light/10 h dark. The larvae were reared on a previously described artificial diet [63].

#### Bioinformatic analysis.

The open reading frames (CDSs) of genes were analyzed at the NCBI (https://www.ncbi.nlm.nih.gov/). The protein sequences were analyzed using MEGA 7 software to construct a phylogenetic tree. The multiple sequence alignment was generated by DNAMAN software (https://lynnon-biosoft-dnaman-eng.software.informer.com/). The protein structural domains were analyzed using SMART (http://smart.embl-heidelberg.de/). The molecular weight and isoelectric point are predicted by ExPASy Compute pI/Mw (https://web.expasy.org/compute_pi/).

#### Gene cloning.

Total RNA was extracted from the larvae and pupae using the TRIzol reagent, after which the RNA concentration was detected. Two micrograms of RNA were used as a template in 6 μl with nuclease-free H_2_O, and 2 μl of AccuRT Reaction Mix (4×), 2 μl of AccuRT Reaction Stopper, and 10 μl of 5 × All-In-One RT Master Mix were mixed in a 4:6 ratio with nuclease-free H_2_O and mixed well to obtain the cDNA template. The primers used were designed (S1 Table), the cDNA was used as a template, the system was prepared, and the program was set up. The amplified PCR products were obtained.

### Quantitative real-time polymerase chain reaction (qRT‒PCR)

Total RNA was isolated from 15 larvae. cDNA was used as a template (1 μl of template, 2 μl of F primer, 2 μl of R primer, and 5 μl of 2 × fluorescent PCR mix). The program was set up to calculate the expression difference using the Ct value given by the instrument. The formula was 2^–(ΔCt sample–ΔCt control)^, and △Ct was the difference between the sample Ct value and the mean value of β-Actin Ct. The primers used were designed as follows (S1 Table).

### Western blot

For total protein extraction, 100 mg of total protein from over three insects the dorsal epidermis, midgut and fat body, 1 mg of brain from over thirty to one hundred individuals at different age groups were washed with 1 × PBS (140 mM NaCl, 2.7 mM KCl, 10 mM Na_2_HPO_4_, and 1.8 mM KH_2_PO_4_), homogenized at low temperature in 40 mM Tris-HCl (pH 8.0) containing 1 mM phenyl methane sulfonyl fluoride (PMSF) (in isopropyl alcohol), and centrifuged at 4°C for 15 min at 12,000 × g. Each sample was subjected to 12.5% SDS‒PAGE with 20 µg of protein, the membrane was transferred to a nitro filter membrane, and the membrane was blocked for 1 h at room temperature with a blocking solution (5% skim milk powder in TBS (10 mM Tris‒HCl, 150 mM NaCl, pH 7.5)). The membrane was incubated overnight at 4°C with diluted primary antibody, washed three times with TBST (TBS with 0.02% Tween) for 10 min/wash, incubated for 2 h at room temperature with secondary antibody (goat anti-rabbit IgG/alkaline phosphatase labeling) diluted 1:5000 in blocking solution, and washed three times with TBST for 10 min/wash. The bands were visualized using the enhanced chemiluminescence (ECL) luminescence method. The immunoreactive protein bands were analyzed via ImageJ software.

### Preparation of rabbit polyclonal antibody

The fragment cDNA of MMP2 was amplified by using the primers Exp-*Mmp2*-F and Exp-*Mmp2*-R and inserted into the plasmid GST at the SacⅠ and SalⅠ sites. Then, the constructs were transformed into *Escherichia coli* (BL21-DE). MMP2 was expressed in the inclusion bodies. MMP2 was purified with a smart Ni NTA beads 6FF column. After renaturation, 1 mg/ml protein was mixed with complete adjuvant at a 1:1 volume ratio and injected subcutaneously into New Zealand white rabbits. After 21 days, the renatured protein was mixed with incomplete adjuvant and injected directly after 14 days. After 7 days, blood was collected, and the supernatant was centrifuged to obtain the MMP2 antibody.

### Commercially purchased antibodies were used in this study

Rabbit polyclonal anti-LC3 antibody was purchased from Abcam (ab109364, Abcam, Cambridge, England), a rabbit monoclonal anti-β-actin antibody (AC026, ABclonal Technology, Wuhan, China), a phospho-histone H3 (Ser10) antibody (9701, Cell Signaling Technology, Danvers, MA, USA), a histone H3 antibody (17168–1-AP, ProteinTech, Beijing, China), and a mouse anti-GFP antibody (AE012, ABclonal Technology, China) were used. Alexa Fluor 488-conjugated sheep anti-rabbit IgG (ab150181, Abcam, Cambridge, England) was used. Horseradish peroxidase (HRP)-labeled sheep anti-rabbit IgG was purchased from AmyJet Scientific, Inc. (Wuhan, China). The antibodies against *H. armigera* MMP2 were prepared in our laboratory.

### Brain whole-mount immunohistochemistry

The brain was dissected and placed in a 96-well plate. One hundred µL of 4% paraformaldehyde was added to each well, and the samples were fixed for 1 h at 4°C and rinsed with PBS for 15 min; this process was repeated four times. Five hundred microliters of blocking solution were added to each well with 0.5% Triton X-100 supplemented with 5% bovine serum albumin (BSA) and blocked at room temperature for 2 h. The blocking solution was aspirated, followed by the addition of primary antibody (1:100), shaking at 4°C overnight for 3 d, flushing with 0.5% Triton X-100 for 15 min, four repeats, the addition of secondary antibody, Alexa Fluor 488-conjugated sheep anti-rabbit IgG, and shaking at room temperature for 2 h. The samples were washed with 0.5% Triton X-100 for 15 min, which was repeated four times, and sealed with 4’,6-diamidino-2-phenylindole (DAPI)-Fluoromount-G fluorescent sealing agent (AmyJet Scientific, Inc., Wuhan, China), laid flat overnight at 4°C, solidified, and then placed upright in the section box. A Zeiss LSM confocal 900 (Germany) was used for imaging.

### Immunohistochemistry

The brain was dissected and fixed in 4% paraformaldehyde at 4°C overnight. The brain was at alcohol gradient dehydration and then put in xylene and Paraffin. The brain was cut into 4 μm sections. The slides were dried at 70°C overnight and subsequently dewaxed with Dewaxing transparent agent and washed with PBS for 5 min; this process was repeated three times. Then the slides were boiled in Sodium Citrate Antigen Retrieval Solution (Solarbio, Beijing, China) for 20 minutes for antigen retrieval. After being washed with PBS for 5 min, this process was repeated three times. 2% Triton X-100 permeabilized for 30 min. The slides were incubated in blocking buffer (5% Bovine serum albumin (BSA) in PBS) for 30 min. The blocking solution was aspirated, followed by the MMP2 antibody (1:50) at 4°C overnight for 2 days, flushing with PBS for 5 min, three repeats, the addition of secondary antibody, Alexa Fluor 488-conjugated sheep anti-rabbit IgG at room temperature for 2 h. The samples were washed with PBS for 5 min, which was repeated three times, and sealed with 4’,6-diamidino-2-phenylindole (DAPI)-Fluoromount-G fluorescent sealing agent (AmyJet Scientific, Inc., Wuhan, China), laid flat at room temperature for 2 h. The images were observed using an Olympus BX51fluorescence microscope (Olympus Optical Co., Tokyo, Japan).

### RNA interference in larvae

dsRNAs were synthesized according to a previous method [64]. Thirty 6th instar-6 h larvae were placed on ice for 15 min, and 5 µl of 0.4 µg/µl dsRNA was injected into the hemolymph via an injection needle forward along the penultimate body segment on the side of the body. A second injection was made from the other side of the larva, 24 h later, from the anterior body segment of the larva at the location of the first injection. The third injection was administered 24 h later. RNA was extracted for subsequent experiments 6 h after the third injection, and protein was extracted for subsequent experiments 24 h after the third injection.

### Hormone stimulation

20-Hydroxyecdysone (20E) (Cayman Chemical, Michigan, United States) was dissolved in dimethyl sulfoxide (DMSO) to 10 mg/ml (20 mM) and then diluted with sterile 1 × PBS to concentrations of 20 ng/µl, 40 ng/µl, 60 ng/µl, and 100 ng/µl. The larvae were subsequently injected with 5 µl of 20E into the hemolymph from the body wall of the first pair of ventral legs of a 6th instar 6 h-old larva (6th–6 h) for 6 h. An equal amount of DMSO was injected as a control. Five microliters of 100 ng/µl 20E were injected into the 6th instar-6 h larva, which was subsequently treated with a time gradient of 1 h, 3 h, 6 h, and 12 h.

### Glucose determination

Two milligrams of brain from about 50–200 pupae or 6–96 h larvae were ground on ice with 20 μL reagents from the Beyotime glucose assay kit (Cat S0201S, Shanghai, China). The sample was then centrifuged at 12000 × g for 10 min. After the reagents were added according to the manufacturer’s instructions, the absorbance at 630 nm was measured by spectrophotometry (Inﬁnite M200PRO NanoQuant, Tecan). A standard curve was created to calculate the glucose levels of the tested samples based on the measured values of the standard samples.

### Glutamate determination

Two milligrams of brain from about 50–200 pupae or 6–96 h larvae were ground on ice with 20 μL reagents from the Mlbio glutamate assay kit (GLU.I-W96-N (1720), Shanghai, China). The sample was then centrifuged at 2000 × g for 10 min. Take the supernatant and add the reagent to adjust the pH value to 9–10. The sample was then centrifuged at 2000 × g for 5 min. After the reagents were added according to the manufacturer’s instructions, the absorbance at 490 nm was measured at 5 min by spectrophotometry (Infinite M200PRO NanoQuant, Tecan). And then add the reagent six, incubate 15 min at room temperature, and the absorbance at 490 nm was measured. A standard curve was created to calculate the glutamate levels of the tested samples based on the measured values of the standard.

### Luciferase assay

The full-length *Foxo* open reading frame was amplified and was inserted into the plasmid pIEx-RFP-His between the enzyme cut sites *Bgl* II and *Sal* Ⅰ; this plasmid was named the FOXO-RFP-His overexpression plasmid. In addition, the 2540 bp 5′ upstream sequence before the start codon of *Mmp2* containing the FOXO binding element (FOXBE) was amplified and inserted into pIEx-4-luciferase-GFP-His plasmid [65] between the *Sma* Ⅰ and *Sac* Ⅰ enzymatic sites by replacing the promoter and enhancer, to generate the *pMmp2*-LUCI-GFP-His reporter plasmid. The *pMmp2*-LUCI-GFP-His and FOXO-RFP-His plasmids were transfected into *H. armigera* epidermal cell lines (HaEpi), and the internal reference plasmid pRL-TK driven by pIE promoter was also transfected together as a luciferase-based control. Luciferase expression levels were detected by western blotting. A Dualucif Firefy and Renilla Assay Kit (UElandy, Suzhou, China) was used according to the manufacturer’s instructions. Luciferase variation was measured with a multimode plate (EnSpire, PerkinElmer, Waltham, USA).

### Statistical analysis

All the experiments were repeated three times. Student′s *t* test was used for paired comparison. Asterisks indicate significant differences (*p < 0.05, **p < 0.01, ***p < 0.001). One-way Analysis of Variance (ANOVA) and two-way ANOVA were used for multiple comparisons with a Bonferroni correction using the software Prism, and different letters indicate significant differences. The bars in the figures represent the mean ± standard deviation (SD) for three separate biological experiments. The protein bands identified via western blot analysis were quantified with ImageJ software.

**Highlights**

1. MMP2 expression is increased in the brain during metamorphosis.
2. MMP2 is specifically distributed in some surface and internal neural cells of the brain.
3. MMP2 prevents autophagy and maintains cell proliferation.
4. MMP2 is required for maintaining the nutrient supply in the brain.
5. 20E via the transcription factor FOXO promotes MMP2 expression.

## Supporting information

S1 Fig Identification of MMPs of *H. armigera.* Red colored MMPs were identified in the *H. armigera,* with the target gene *Mmp2* marked by the red box. *H. armigera*: *Helicoverpa armigera*; *B. mori*: *Bombyx mori*; *D. melanogaster*:*Drosophila melanogaster; A. aegypti*:*Aedes aegypti*; *H. sapiens*:*Homo sapiens*; *M. musculus*: *Mus musculus*. A phylogenetic tree of MMPs in *H. armigera* was analyzed by MAGE7.
(DOCX)

S2 Fig Analysis of MMPs structural domains by SMART. ZnMc is a Zinc-dependent metalloprotease domain. The blue box indicates the transmembrane region. HX is a hemopexin-like repeat. The black box represents the peptidoglycan binding domain (PGBD) and the DUF domain of membrane matrix metalloproteinases in *H. sapiens*.
(DOCX)

S3 Fig Alignment of the MMPs of *H. armigera* by DNAMAN. The sequence in red boxes was used for preparing antibodies. The sequence in the orange box is the interference sequence.
(DOCX)

S4 Fig Hemi heat map of MMPs in *H. armigera.* The original data were in S2 Table. 6th-72 h wing D: 6th-72 h wing disc.
(DOCX)

S5 Fig Screening of MMPs differentially expressed in the brain. qPCR detected the expression of the three *Mmps* during the feeding and metamorphosis stages, and selected 6th-24 h for the feeding stage and 6th-96 h for the metamorphosis stage. The bars indicate the means ± SD from three biological experiments and three technical repeats. Statistical analyses were conducted using Student′s *t*-test (***, *p* < 0.001).
(DOCX)

S6 Fig Specificity of antibodies against MMP2. (A) SDS-PAGE to show MMP2 antigen (21 kDa) fused with GST tag (26 kDa) and overexpressed in *E. coli*. (B) Specificity of antibodies against MMP2 by western blot to detect MMP2 in 6th-96 h brain. The gel concentration was 12.5%. M: molecular markers.
(DOCX)

S7 Fig Localization of MMP2 in the brain by Whole-Mount (Z-projection) and single slide by Confocal. The green fluorescence indicated the MMP2 stained with antibodies. The brain was from the 6th-96 h larva.
(DOCX)

S8 Fig Localization of MMP2 in the brain by immunohistochemistry. The slides were from immunohistochemistry. The green fluorescence indicated the MMP2 stained with antibodies. The brain was from the 6th-96 h larva.
(DOCX)

S9 Fig Glutamate and glucose levels in the hemolymph. (A) Glucose levels in the hemolymph at different developmental stages. (B) and (C) Glucose levels in the larval hemolymph and pupal hemolymph after the last injection of *dsMmp2* into the 6th-instar 6 h larval hemocoel. (D) Glutamate levels in the hemolymph at different developmental stages. (E) and (F) Glutamate levels in the larval hemolymph and pupal hemolymph after the last injection of *dsMmp2* into the 6th-instar 6 h larval hemocoel. All the experiments were repeated three times. The bars indicate the means ± SD. Statistical analyses were conducted using Student′s *t* test (*, *p* < 0.05, **, *p* < 0.01, ***, *p* < 0.001).
(DOCX)

S10 Fig Transcriptome analysis of the expression of the transporters. The original data were in S3 Table. 6th-72 h wing D: 6th-72 h wing disc.
(DOCX)

S11 Fig Expression levels of transporters after *Mmp2* knockdown. The samples were taken from the 6th instar 96 h larvae. The developmental time was controlled by waiting for an additional 20 h to take samples from the *Mmp2* knockdown group than the control group after dsRNA injection. All the experiments were repeated three times using three preparations of RNA and cDNA. The bars indicate the means ± SD. Statistical analyses were conducted using Student′s *t* test (***, *p* < 0.001).
(DOCX)

S1 Table Primers used in the experiments. (DOCX)

S2 Table The expression levels of MMPs by the heatmap analysis. (DOCX)

S3 Table The expression of the transporters in the heatmap. (DOCX)

S1 Data The original data for the statistical analysis in this study. Fig 1Ai protein. Fig 1B mRNA. Fig 3Ai Brain size. Fig 3B Pupation time. Fig 3D Phenotype. Fig 3E Efficacy of RNAi. Fig 3Fi + Ii-LC3. Fig 3Gi + Ji-P-H3. Fig 3H-gene expression. Fig 3K-gene expression. Fig 4A Glucose. Fig 4B and C Glucose dsMmp2. Fig 4D Glytamate. Fig 4E + 4F Glytamate dsMmp2. Fig 5A transporter expression. Fig 5B dsMMP2-transporter expression. Fig 6 Glucose Glu dsMmp2. Fig 7A-20E dose. Fig 7B 20E time. Fig 7C dsEcr. Fig 7D dsFOXO. Fig 7G LUCI-activity. Fig 7H LUCI-WB.
(ZIP)
