# Supplementary material for: 20-hydroxyecdysone promotes brain development via upregulating MMP2 expression during metamorphosis in Helicoverpa armigera
Source: PLoS Genet. 2026 Jan 22;22(1):e1012032. doi: 10.1371/journal.pgen.1012032 (PMC12858071; doi:10.1371/journal.pgen.1012032)
Supplement: S1 Fig — Red colored MMPs were identified in the H. armigera, with the target gene Mmp2 marked by the red box. H. armigera: Helicoverpa armigera; B. mori: Bombyx mori; D. melanogaster:Drosophila melanogaster; A. aegypti:Aedes aegypti; H. sapiens:Homo sapiens; M. musculus: Mus musculus. A phylogenetic tree of MMPs in H. armigera was analyzed by MAGE7. (DOCX) [file pgen.1012032.s001.docx]

**
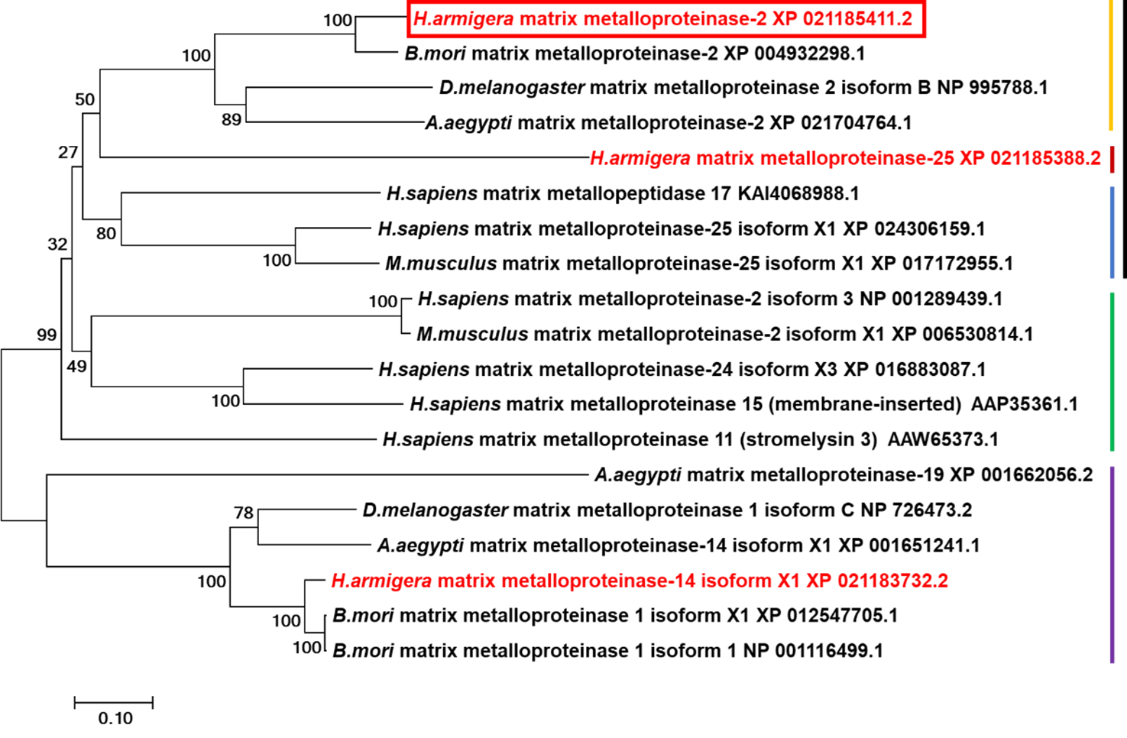
**

**S1 Fig.** **Identification of MMPs of *H. armigera*.** Red colored MMPs were identified in the *H. armigera,* with the target gene *Mmp2* marked by the red box. *H. armigera*：*Helicoverpa armigera*；*B. mori*：*Bombyx mori*；*D. melanogaster*：*Drosophila melanogaster；A. aegypti*：*Aedes aegypti*; *H. sapiens*：*Homo sapiens*；*M. musculus*：*Mus musculus*. A phylogenetic tree of MMPs in *H. armigera* analyzed by MAGE7.
