# Supplementary material for: 20-hydroxyecdysone promotes brain development via upregulating MMP2 expression during metamorphosis in Helicoverpa armigera
Source: PLoS Genet. 2026 Jan 22;22(1):e1012032. doi: 10.1371/journal.pgen.1012032 (PMC12858071; doi:10.1371/journal.pgen.1012032)
Supplement: S2 Fig — ZnMc is a Zinc-dependent metalloprotease domain. The blue box indicates the transmembrane region. HX is a hemopexin-like repeat. The black box represents the peptidoglycan binding domain (PGBD) and the DUF domain of membrane matrix metalloproteinases in H. sapiens. (DOCX) [file pgen.1012032.s002.docx]

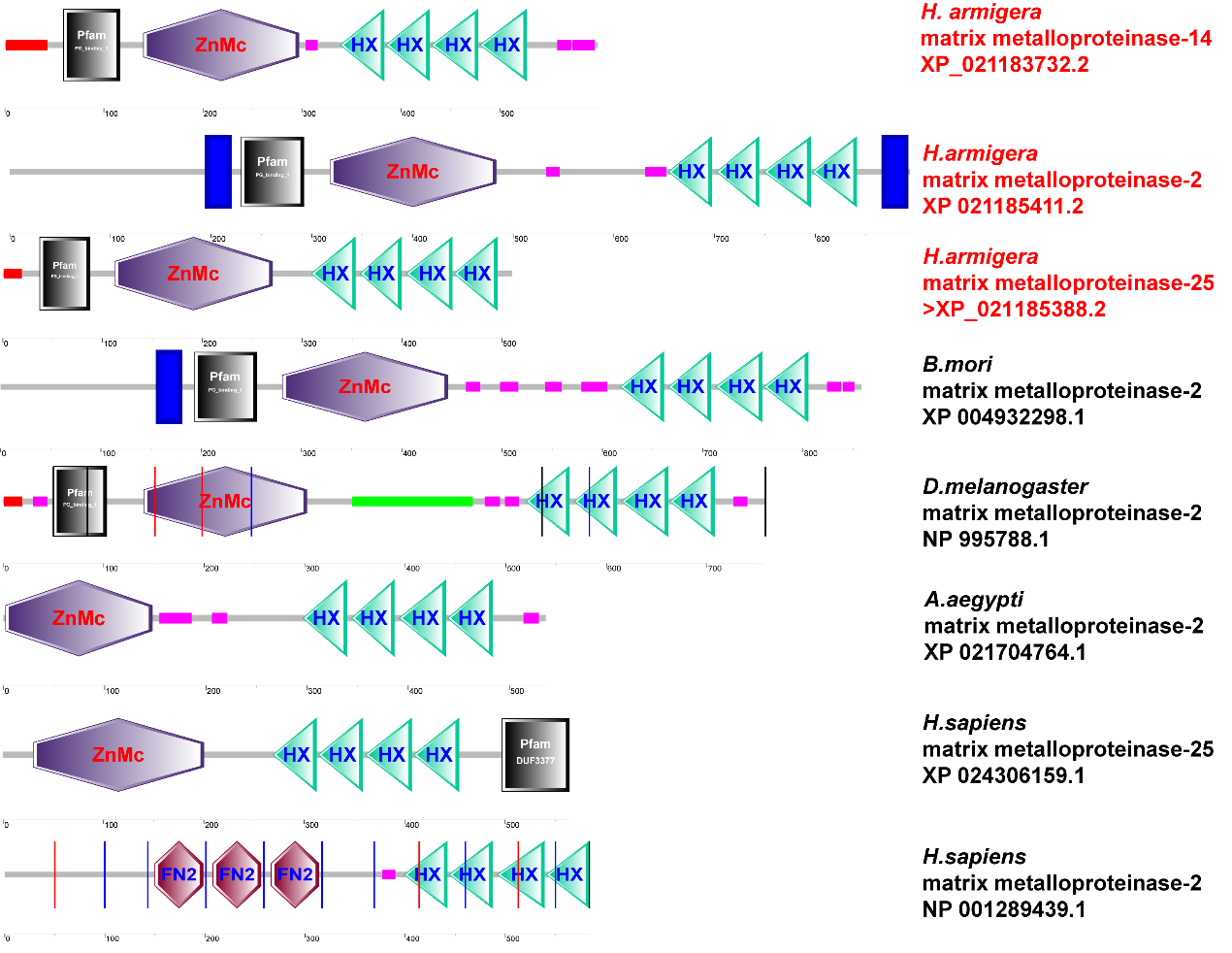


**S2 Fig.** **Analysis of MMPs structural domains by SMART.** ZnMc is a Zinc-dependent metalloprotease domain. The blue box indicates the transmembrane region. HX is a hemopexin-like repeat. The black box represents the peptidoglycan binding domain (PGBD) and the DUF domain of membrane matrix metalloproteinases in *H. sapiens*.
