# Supplementary material for: 20-hydroxyecdysone promotes brain development via upregulating MMP2 expression during metamorphosis in Helicoverpa armigera
Source: PLoS Genet. 2026 Jan 22;22(1):e1012032. doi: 10.1371/journal.pgen.1012032 (PMC12858071; doi:10.1371/journal.pgen.1012032)
Supplement: S3 Fig — The sequence in red boxes was used for preparing antibodies. The sequence in the orange box is the interference sequence. (DOCX) [file pgen.1012032.s003.docx]

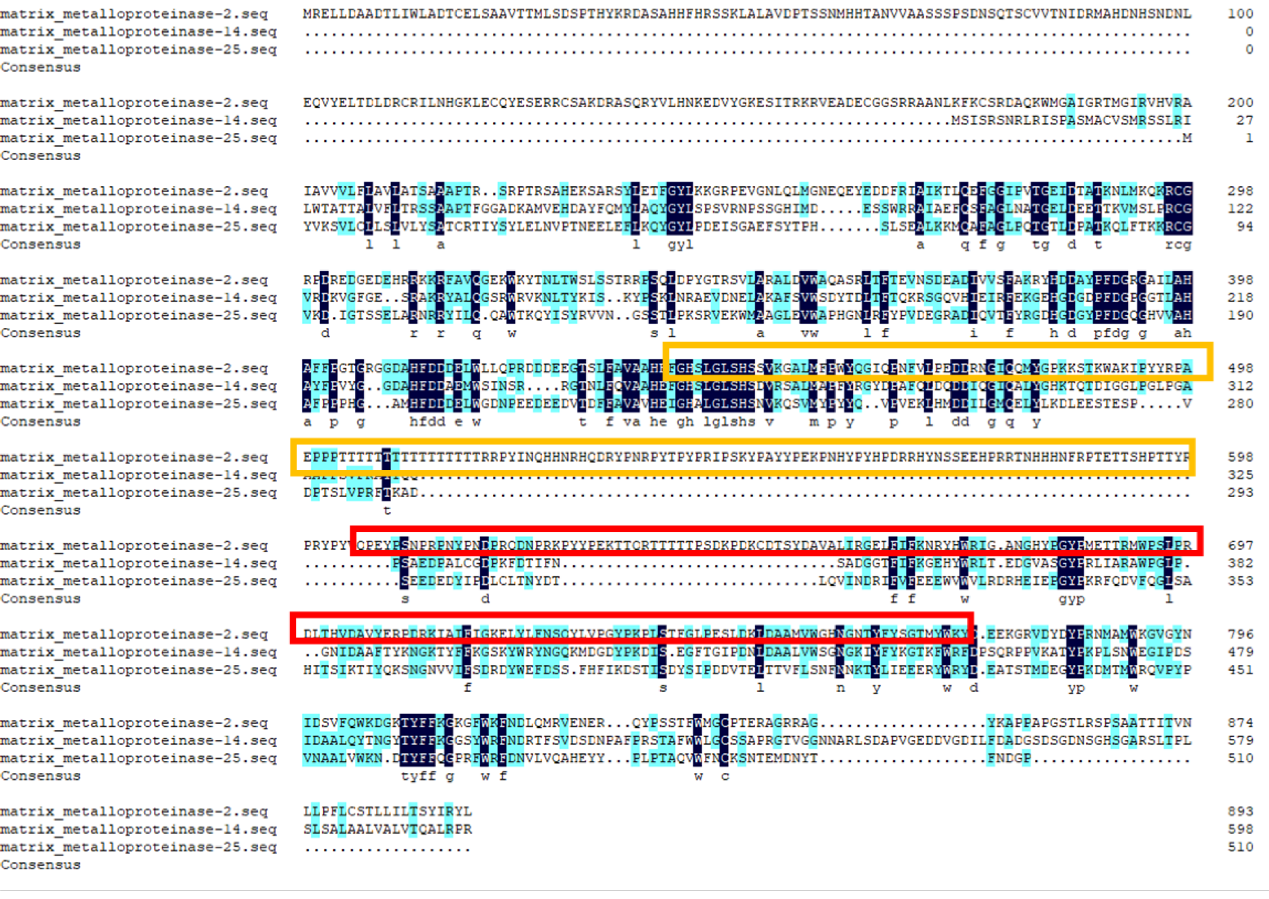


**S3 Fig. Alignment of the MMPs of *H. armigera* by DNAMAN.** The sequence in red boxes was used for preparing antibodies. The sequence in the orange box is the interference sequence.
