# Supplementary figures and images for: 20-hydroxyecdysone promotes brain development via upregulating MMP2 expression during metamorphosis in Helicoverpa armigera
Source: PLoS Genet. 2026 Jan 22;22(1):e1012032. doi: 10.1371/journal.pgen.1012032 (PMC12858071; doi:10.1371/journal.pgen.1012032)

**
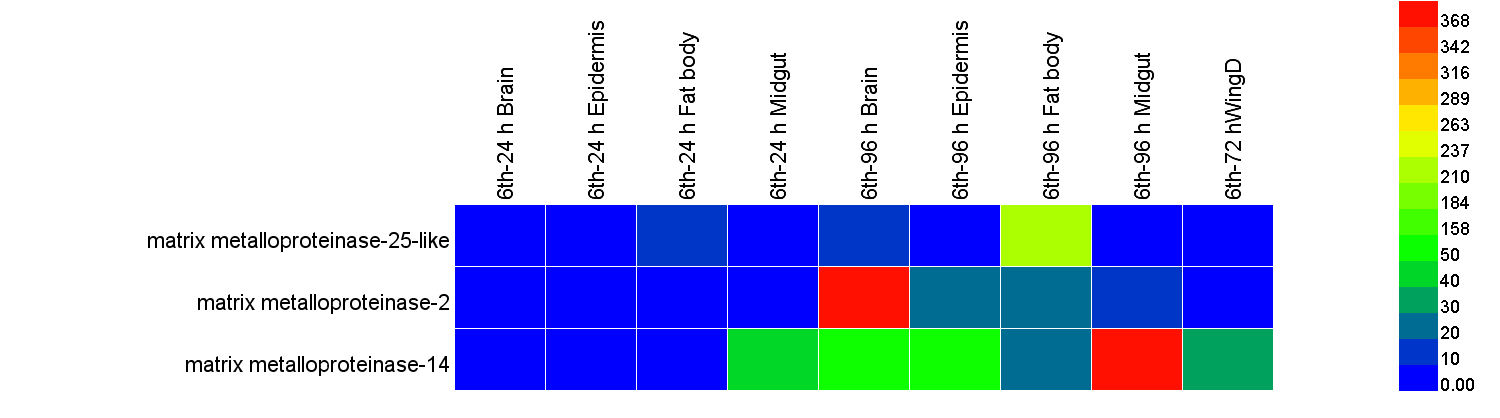
**

**S4 Fig. Hemi heat map of MMPs in *H. armigera.***The original data was in Table S2. 6th-72 h wingD: 6th-72 h wing disc.

Supplement: S4 Fig — The original data were in S2 Table. 6th-72 h wing D: 6th-72 h wing disc. (DOCX) [file pgen.1012032.s004.docx]
