# Supplementary material for: 20-hydroxyecdysone promotes brain development via upregulating MMP2 expression during metamorphosis in Helicoverpa armigera
Source: PLoS Genet. 2026 Jan 22;22(1):e1012032. doi: 10.1371/journal.pgen.1012032 (PMC12858071; doi:10.1371/journal.pgen.1012032)
Supplement: S5 Fig — The bars indicate the means ± SD from three biological experiments and three technical repeats. Statistical analyses were conducted using Student′s t-test (***, p < 0.001). (DOCX) [file pgen.1012032.s005.docx]

**
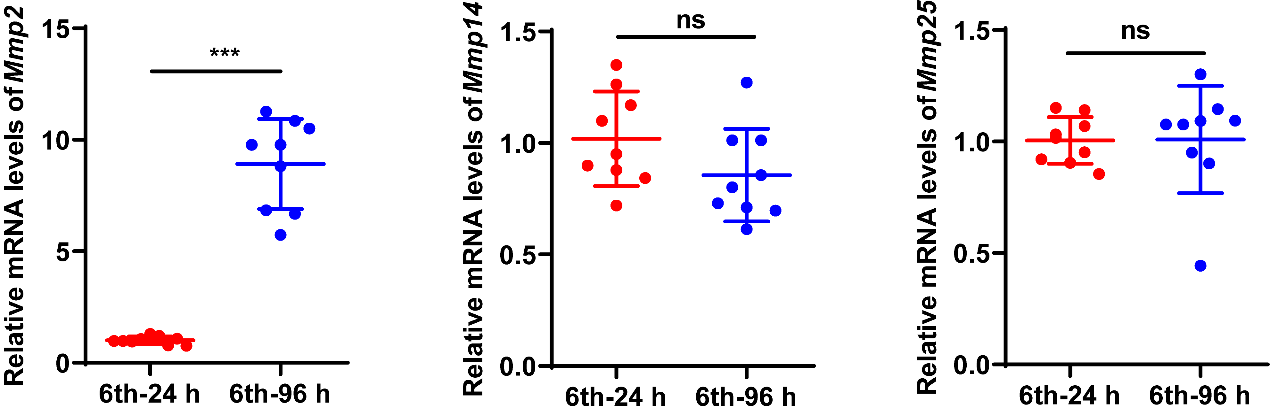
**

**S5 Fig. Screening of MMPs differentially expressed in the brain.** qPCR detected the expression of the three *Mmps* during the feeding and metamorphosis stages, and selected 6th-24 h for the feeding stage and 6th-96 h for the metamorphosis stage. The bars indicate the means ± SD from three biological experiments and three technical repeats. Statistical analyses were conducted using Student′s *t*-test (***, *p* < 0.001).
