# Supplementary material for: 20-hydroxyecdysone promotes brain development via upregulating MMP2 expression during metamorphosis in Helicoverpa armigera
Source: PLoS Genet. 2026 Jan 22;22(1):e1012032. doi: 10.1371/journal.pgen.1012032 (PMC12858071; doi:10.1371/journal.pgen.1012032)
Supplement: S6 Fig — (A) SDS-PAGE to show MMP2 antigen (21 kDa) fused with GST tag (26 kDa) and overexpressed in E. coli. (B) Specificity of antibodies against MMP2 by western blot to detect MMP2 in 6th-96 h brain. The gel concentration was 12.5%. M: molecular markers. (DOCX) [file pgen.1012032.s006.docx]

**
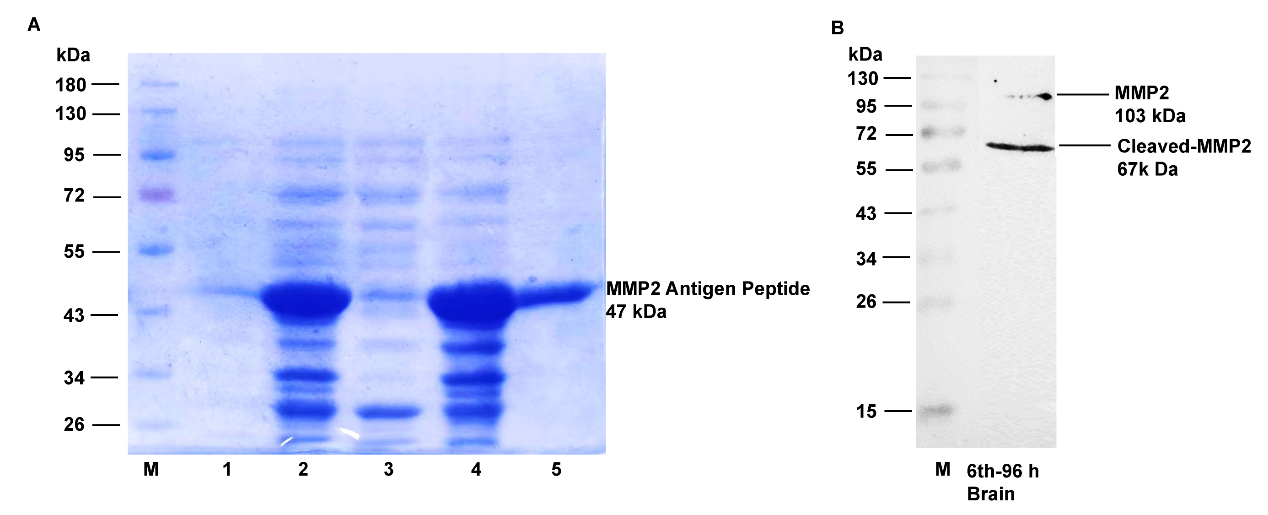
**

**S6 Fig. Specificity of antibodies against MMP2.** (A) SDS-PAGE to show MMP2 antigen (21 kDa) fused with GST tag (26 kDa) and overexpressed in *E. coli*. (B) Specificity of antibodies against MMP2 by western blot to detect MMP2 in 6th-96 h brain. The gel concentration was 12.5%. M: molecular markers.
