# Supplementary material for: 20-hydroxyecdysone promotes brain development via upregulating MMP2 expression during metamorphosis in Helicoverpa armigera
Source: PLoS Genet. 2026 Jan 22;22(1):e1012032. doi: 10.1371/journal.pgen.1012032 (PMC12858071; doi:10.1371/journal.pgen.1012032)
Supplement: S7 Fig — The green fluorescence indicated the MMP2 stained with antibodies. The brain was from the 6th-96 h larva. (DOCX) [file pgen.1012032.s007.docx]

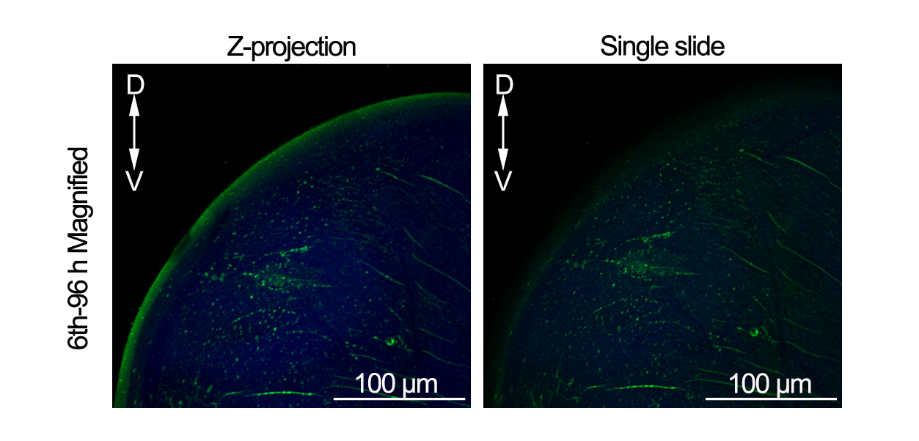


**S7 Fig.** **Localization of MMP2 in the brain by** **Whole-Mount (Z-projection) and single slide by Confocal.** The green fluorescence indicated the MMP2 stained with antibodies. The brain was from the 6th-96 h larva.
