# Supplementary material for: 20-hydroxyecdysone promotes brain development via upregulating MMP2 expression during metamorphosis in Helicoverpa armigera
Source: PLoS Genet. 2026 Jan 22;22(1):e1012032. doi: 10.1371/journal.pgen.1012032 (PMC12858071; doi:10.1371/journal.pgen.1012032)
Supplement: S8 Fig — The slides were from immunohistochemistry. The green fluorescence indicated the MMP2 stained with antibodies. The brain was from the 6th-96 h larva. (DOCX) [file pgen.1012032.s008.docx]

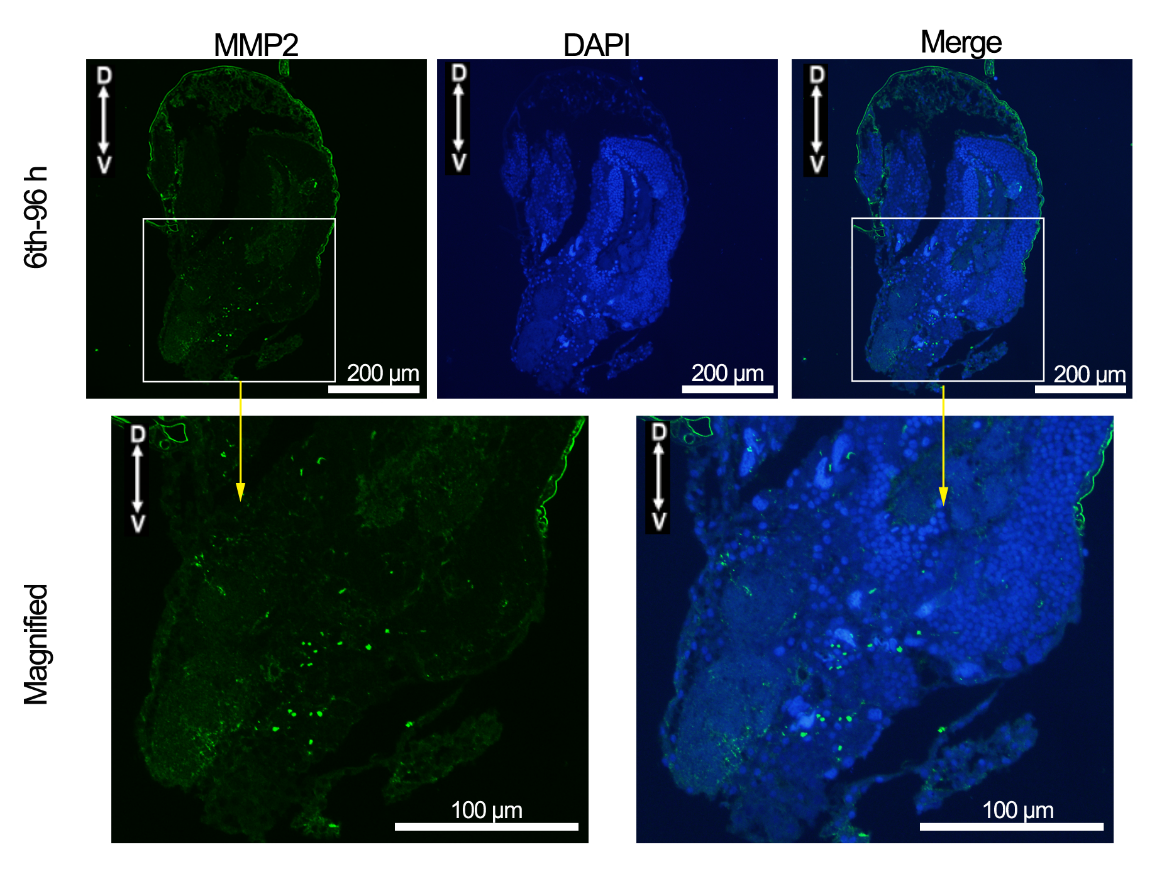


**S8 Fig. Localization of MMP2 in the brain.** The slides were from immunohistochemistry. Half brain was showed. The green fluorescence indicated the MMP2 stained with antibodies. The brain was from the 6th-96 h larva.
