# Supplementary material for: 20-hydroxyecdysone promotes brain development via upregulating MMP2 expression during metamorphosis in Helicoverpa armigera
Source: PLoS Genet. 2026 Jan 22;22(1):e1012032. doi: 10.1371/journal.pgen.1012032 (PMC12858071; doi:10.1371/journal.pgen.1012032)
Supplement: S9 Fig — (A) Glucose levels in the hemolymph at different developmental stages. (B) and (C) Glucose levels in the larval hemolymph and pupal hemolymph after the last injection of dsMmp2 into the 6th-instar 6 h larval hemocoel. (D) Glutamate levels in the hemolymph at different developmental stages. (E) and (F) Glutamate levels in the larval hemolymph and pupal hemolymph after the last injection of dsMmp2 into the 6th-instar 6 h larval hemocoel. All the experiments were repeated three times. The bars indicate the means ± SD. Statistical analyses were conducted using Student′s t test (*, p < 0.05, **, p < 0.01, ***, p < 0.001). (DOCX) [file pgen.1012032.s009.docx]

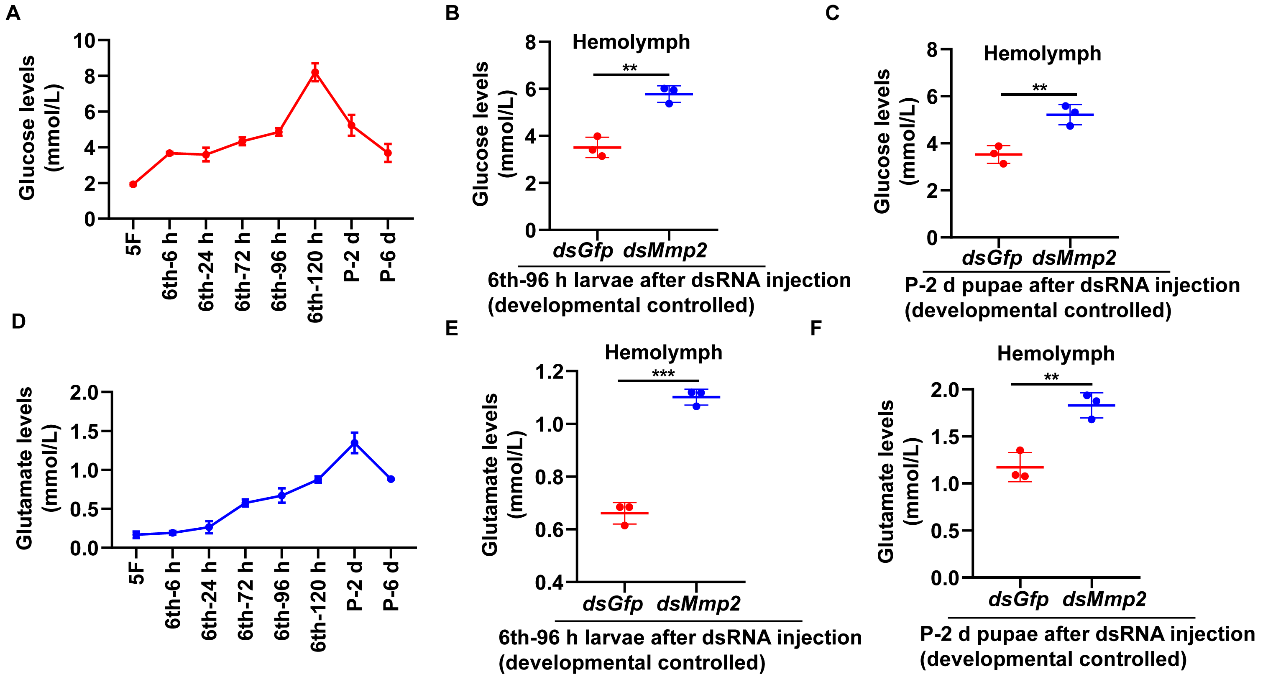


**S9 Fig.** **Glutamate and glucose levels in the hemolymph.** (A) Glucose levels in the hemolymph at different developmental stages. (B) and (C) Glucose levels in the larval hemolymph and pupal hemolymph after the last injection of *dsMmp2* into the 6th-instar 6 h larval hemocoel. (D) Glutamate levels in the hemolymph at different developmental stages. (E) and (F) Glutamate levels in the larval hemolymph and pupal hemolymph after the last injection of *dsMmp2* into the 6th-instar 6 h larval hemocoel. All the experiments were repeated three times. The bars indicate the means ± SD. Statistical analyses were conducted using Student′s *t* test (*, *p* < 0.05, **, *p* < 0.01, ***, *p* < 0.001).
