# Supplementary material for: 20-hydroxyecdysone promotes brain development via upregulating MMP2 expression during metamorphosis in Helicoverpa armigera
Source: PLoS Genet. 2026 Jan 22;22(1):e1012032. doi: 10.1371/journal.pgen.1012032 (PMC12858071; doi:10.1371/journal.pgen.1012032)
Supplement: S10 Fig — The original data were in S3 Table. 6th-72 h wing D: 6th-72 h wing disc. (DOCX) [file pgen.1012032.s010.docx]

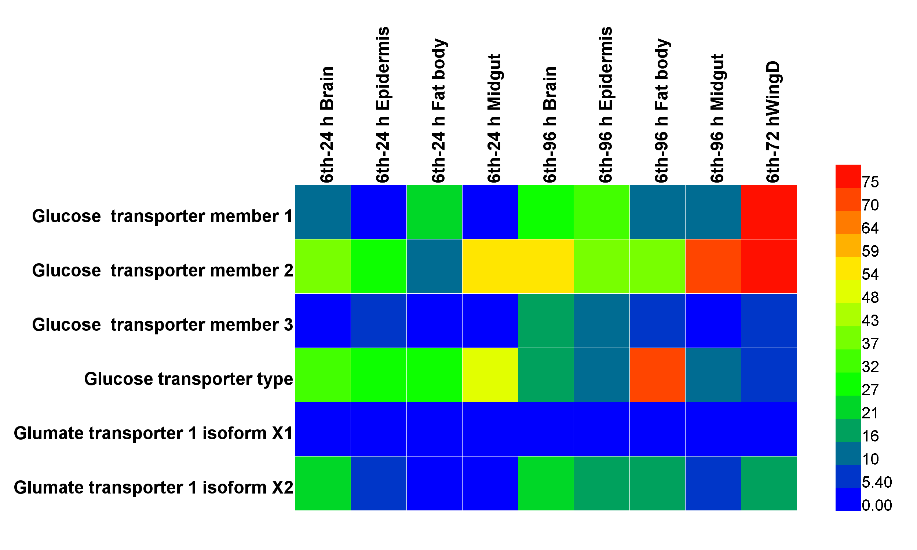


**S10 Fig. Transcriptome analysis of the expression of the transporters.** The original data was in Table S3. 6th-72 h wingD: 6th-72 h wing disc.
