# Supplementary material for: 20-hydroxyecdysone promotes brain development via upregulating MMP2 expression during metamorphosis in Helicoverpa armigera
Source: PLoS Genet. 2026 Jan 22;22(1):e1012032. doi: 10.1371/journal.pgen.1012032 (PMC12858071; doi:10.1371/journal.pgen.1012032)
Supplement: S11 Fig — The samples were taken from the 6th instar 96 h larvae. The developmental time was controlled by waiting for an additional 20 h to take samples from the Mmp2 knockdown group than the control group after dsRNA injection. All the experiments were repeated three times using three preparations of RNA and cDNA. The bars indicate the means ± SD. Statistical analyses were conducted using Student′s t test (***, p < 0.001). (DOCX) [file pgen.1012032.s011.docx]

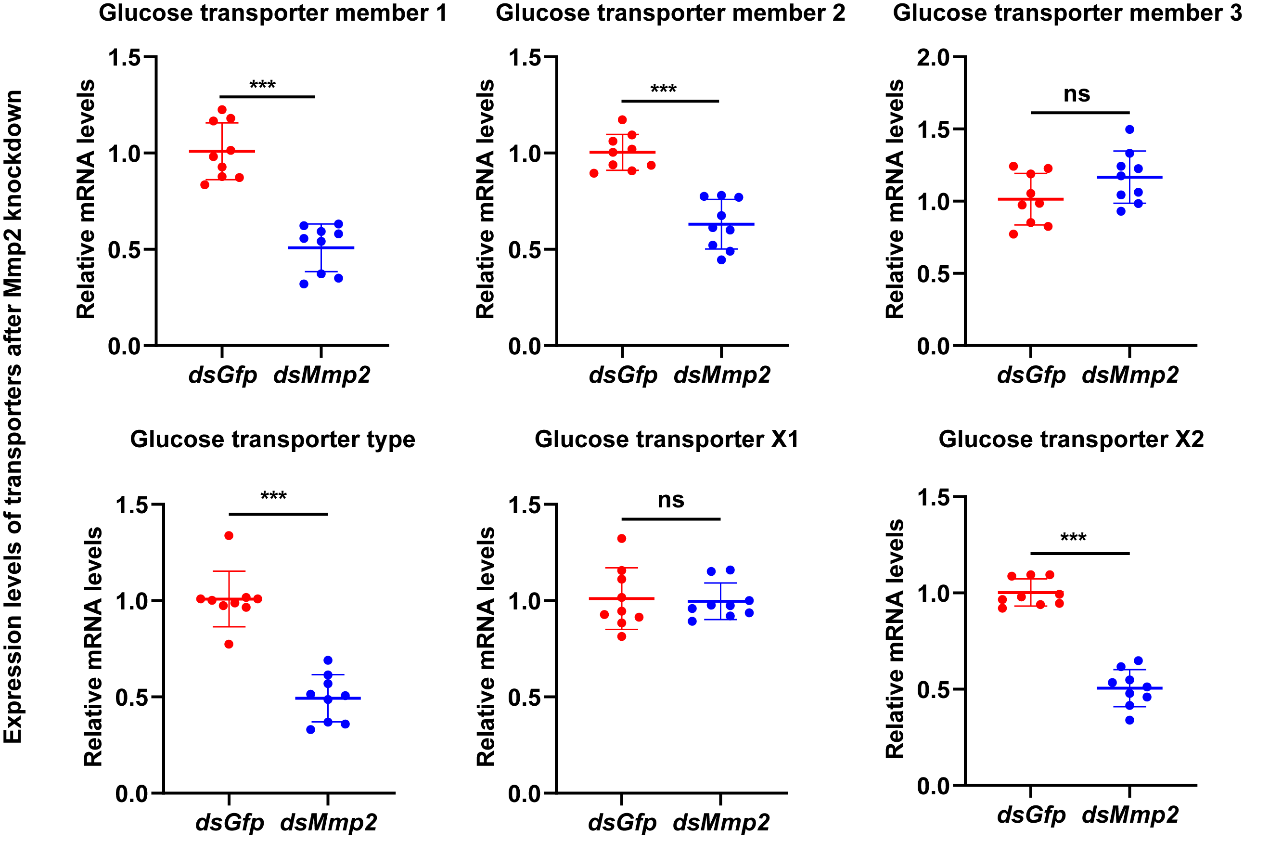


**S11 Fig. Expression levels of transporters after *Mmp2* knockdown.** The samples were taken from the 6th instar 96 h larvae. The developmental time was controlled by waiting for additional 20 h to take samples from the *Mmp2* knockdown group than the control group after dsRNA injection. All the experiments were repeated three times using three preparations of RNA and cDNA. The bars indicate the means ± SD. Statistical analyses were conducted using Student′s *t* test (***, *p* < 0.001).
