# Supplementary material for: 20-hydroxyecdysone promotes brain development via upregulating MMP2 expression during metamorphosis in Helicoverpa armigera
Source: PLoS Genet. 2026 Jan 22;22(1):e1012032. doi: 10.1371/journal.pgen.1012032 (PMC12858071; doi:10.1371/journal.pgen.1012032)
Supplement: S1 Table — (DOCX) [file pgen.1012032.s012.docx]

**S1 Table Primers used in the experiments.**

| Primer name | Sequence (5’-3’) |
| --- | --- |
| Overexpression |  |
| Exp-*Mmp2*-F | TACTCAGGATCCTACCCGAGCAATCCCAGACC |
| Exp-*Mmp2*-R(Fig S6) | TACTCAGTCGACCAACACGACCTTTTTCCTCAT |
| qRT-PCR |  |
| *Mmp2*-RTF | AAAGAGGGTTGAGGCTGACG |
| *Mmp2*-RTR | AACAACACCACGACGGCTAT |
| *Mmp14-*RTF | ACAACCTGGACGCTGCTTTA |
| *Mmp14*-RTR | GCGCCAGAACTTAGTACCCT |
| *Mmp25-*RTF | ATGGACCTTGAGCCTTGTCG |
| *Mmp25*-RTR | TTTGAGGCGGAGTTTGGTGA |
| *c-Myc*-RTF | ATGAAGACTGGAGGATAATGGA |
| *c-Myc*-RTR | TGTTGTGGGTGTAGTGGTTG |
| *Wnt*-RTF | GTCACGGTATGTCAGGCTCG |
| *Wnt*-RTR | GGCTCCAGGTCAGTATTAGGC |
| *Atg8-* RTF | GAAGAGAAAGACCGAAGGCG |
| *Atg8-* RTR | TCAGGTCGTAGATGTATGCGT |
| *β-actin*-RTF | CCTGGTATTGCTGACCGTATGC |
| *β-actin*-RTR | CTGTTGGAAGGTGGAGAGGGAA |
| *Glut-1-* RTF | CGGCAGGTTTATCATTGGCG |
| *Glut-1-* RTR | GACCAATCCAAATGCCACCG |
| *Glut-2-* RTF | TTTGGAGATCCGAGCAACCT |
| *Glut-2-* RTR | AGGTTTGTACGTGCAGGAGTT |
| *Glut-3-* RTF | TGCTGCGTTTACTTTAGGCG |
| *Glut-3-* RTR | TCCAAACGACTTAAGCGCCA |
| *Glut-t-* RTF | CACGGGAGCTCGTTGAAGTA |
| *Glut-t-* RTR | CGGCACGGGAAGTAGCATAG |
| *Gt-X1-*RTF | CAGCACACAAAGAGAGCAGT |
| *Gt-X1-*RTR | TGAAGAACCCGAAGAATGCCA |
| *Gt-X2-*RTF | TATGGACGGGAAATGAAGAGCA |
| *Gt-X2-*RTR | CGAAGAATGCCAGTATCGCC |
| *RNAi* |  |
| *Mmp2*-RNAiF | GCGTAATACGACTCACTATAGGACCGCAATGGTATCCAGCAA |
| *Mmp2*-RNAiR | GCGTAATACGACTCACTATAGGGTGGTGGTCGTTGTTCGTTG |
| *Gfp*-RNAiF | GCGTAATACGACTCACTATAGGGATGGTCCCAATTCTCGTGGAAC |
| *Gfp*-RNAiR | GCGTAATACGACTCACTATAGGGACTTGAAGTTGACCTTGATGCC |
| *Foxo*-T7F | GCGTAATACGACTCACTATAGGCAAGACAACAGACTCACG |
| *Foxo*-T7R | GCGTAATACGACTCAETATAGGTTGTCCGAAGTCCGTTTG |
| *Ecr-*RNAi | GCGTAATACGACTCACTATAGGGACGCTGGTATAACAACGGAGGA |
| *Ecr*-RNAi | GCGTAATACGACTCACTATAGGGAAGCTGGAGACAACTCCTCACG |
| *Glut-1-* RNAiF | GCGTAATACGACTCACTATAGGGTCGGACTGTGTGTCGCTAA |
| *Glut-1-* RNAiR | GCGTAATACGACTCACTATAGGACTGTCATGCACCTTAACTCG |
| *Glut-2-* RNAiF | GCGTAATACGACTCACTATAGGTTGTGGTGATTGTAATGACGTGT |
| *Glut-2-* RNAiR | GCGTAATACGACTCACTATAGGATGTCGTGATCTCCCGAAGG |
| *Glut-t-* RNAiF | GCGTAATACGACTCACTATAGGGACATGCACGTTGAAGACCG |
| *Glut-t-* RNAiR | GCGTAATACGACTCACTATAGGCGAACAGCTCCGACAACAAC |
| *Gt-X2-*RNAiF | GCGTAATACGACTCACTATAGGCTCAAAGATCCGTCACCCGT |
| *Gt-X2-*RNAiR | GCGTAATACGACTCACTATAGGAGGTAGCGATGGTGTTGGAC |
| Luci |  |
| *Foxo-F* | TACTCAGAATTCTTGGAACCTTTGGGCGAG |
| *Foxo-R* | TACTCACTCGAGCTACATGGAATCGGCGAGAGA |

**The GenBank numbers corresponding to the genes in the table.** *Mmp2*: XP021185411.2; *c-Myc*: AHN95658.1; *Wnt*: AHN95659.1; *β-actin*: ALP73405.1. *Mmp14:*XP 021183732.2*; Mmp25:* XP 021185388.2; *Atg8:* XM_021325898.1；*Foxo*: *XM_021330987.1;* *Ecr*: ACD74807.1; *Glut-1*: XM_021337989.1; *Glut-2*: XM_021335166.1; *Glut-3*: XM_021325280.1; *Glut-t*: XM_021333383.1; *Gt-X1*: XM_021328019.1; *Gt-X2*: XM_021337501.1. *Foxo*: XM_021330987.1;
