# Supplementary material for: 20-hydroxyecdysone promotes brain development via upregulating MMP2 expression during metamorphosis in Helicoverpa armigera
Source: PLoS Genet. 2026 Jan 22;22(1):e1012032. doi: 10.1371/journal.pgen.1012032 (PMC12858071; doi:10.1371/journal.pgen.1012032)
Supplement: S2 Table — (DOCX) [file pgen.1012032.s013.docx]

**S2 Table The expression levels of MMPs in the heatmap.**

| Nr | 6th-24 h Brain | 6th-24 h Epidermis | 6th-24 h Fat body | 6th-24 h Midgut | 6th-96 h Brain | 6th-96 h Epidermis | 6th-96 h Fat body | 6th-96 h Midgut | 6th-72 h  WingD |
| --- | --- | --- | --- | --- | --- | --- | --- | --- | --- |
| matrix metalloproteinase-25-like | 0 | 1 | 18 | 0 | 11 | 8 | 217 | 3 | 3 |
| matrix metalloproteinase-2 | 8 | 3 | 3 | 1 | 130 | 21 | 28 | 16 | 9 |
| matrix metalloproteinase-14 | 4 | 2 | 8 | 49 | 377 | 103 | 26 | 395 | 34 |
