# Supplementary material for: 20-hydroxyecdysone promotes brain development via upregulating MMP2 expression during metamorphosis in Helicoverpa armigera
Source: PLoS Genet. 2026 Jan 22;22(1):e1012032. doi: 10.1371/journal.pgen.1012032 (PMC12858071; doi:10.1371/journal.pgen.1012032)
Supplement: S3 Table — (DOCX) [file pgen.1012032.s014.docx]

**S3 Table The expression of the transporters in the heatmap.**

| Nr | 6th-24 h Brain | 6th-24 h Epidermis | 6th-24 h Fat body | 6th-24 h Midgut | 6th-96 h Brain | 6th-96 h Epidermis | 6th-96 h Fat body | 6th-96 h Midgut | 6th-96 h WingD |
| --- | --- | --- | --- | --- | --- | --- | --- | --- | --- |
| Glucose transporter member 1 | 10 | 4 | 24 | 1 | 31 | 36 | 10 | 14 | 81 |
| Glucose transporter member 2 | 39 | 28 | 11 | 57 | 58 | 40 | 39 | 71 | 76 |
| Glucose transporter member 3 | 3 | 6 | 3 | 0 | 16 | 12 | 8 | 5 | 8 |
| Glucose transporter type | 34 | 27 | 27 | 52 | 16 | 10 | 71 | 14 | 6 |
| Glutamate transporter 1 isoform X1 | 1 | 0 | 0 | 0 | 1 | 0 | 0 | 0 | 0 |
| Glutamate transporter 1 isoform X2 | 23 | 6 | 1 | 2 | 22 | 18 | 20 | 6 | 16 |
